# Human atrial skinned muscle fibers exhibit reduced length-dependent activation but show faster force development kinetics than ventricular muscle

**DOI:** 10.1016/j.yjmcc.2025.12.001

**Published:** 2025-12-04

**Authors:** Alexandre Lewalle, Gregory Milburn, Jania Bell, Kenneth S. Campbell, Steven A. Niederer

**Affiliations:** a National Heart and Lung Institute, Faculty of Medicine, Imperial College London, London, United Kingdom; b Department of Physiology, University of Kentucky, Lexington, KY, United States of America; c Division of Cardiovascular Medicine, University of Kentucky, Lexington, KY, United States of America

**Keywords:** Length-dependent activation, Bayesian calibration, Human atrial cardiomyocytes, Cardiomyocyte contraction, Frank–Starling mechanism, Biophysical modeling

## Abstract

In humans, the left atria (LA) and the left ventricle (LV) play distinct physiological roles and express sarcomeric proteins with chamber-specific patterns. Despite these important differences, most multi-chamber descriptions of the heart assume uniform myocardial properties. To facilitate a more accurate representation of cardiac function, we measured and compared the contractile properties of isolated skinned human LA and LV muscle fibers at 37 °C. Our experimental measurements included the length-dependent activation (LDA) of force in the isometric steady state, the force response to small quick length changes, and tension redevelopment dynamics. The LV measurements display more pronounced LDA behavior compared to LA, whereas the LA dynamics is generally faster than LV.

To elucidate these differences mechanistically, we used the LA and LV experimental datasets to fit a biophysical model framework to produce a representative model for each chamber. Our Bayesian statistical approach aimed to maximize the objectivity of the model calibrations and to allow a systematic assessment of chamber-specific parameter differences. Passive mechanical properties emerge as the principal determinant of LDA behavior. However, variations in cross-bridge cycling kinetics account more significantly for LA/LV differences in the ATP consumption to produce a given isometric force.

These results constitute the first systematic biophysical comparison of LA and LV cardiomyocyte contraction mechanics in humans, paving the way to further investigation of their roles within the broader cardiovascular physiological context.

## 1. Introduction

The heart is a dynamic biomechanical system whose chambers contract and relax in closely regulated coordination [1–3]. The atria and ventricles, while sharing many generic features of muscle contraction, achieve this by performing different functional roles. For instance, the atrial walls must contract rapidly during the “booster pump” phase of the atrial cycle to inject blood into the expanding ventricles [2,4]. They then relax promptly to allow refilling and to facilitate ventricular contractions, which must sustain prolonged force against large afterloads. A quantitative characterization of cellular contraction is essential for achieving a thorough understanding of cardiac function and, ultimately, identifying mechanistic causes of cardiovascular disease. However, we are not aware of any existing biophysical comparisons based on isolated tissue samples from humans.

Clinical and experimental evidence highlights notable differences between atrial and ventricular muscle mechanical properties and behavior, often studied in animal models. These include the Frank-Starling effect (whereby the contraction force increases with tissue stretch) [4,5], myosin cross-bridge kinetics [6,7], sarcomere morphologies [8], and differential expression of sarcomeric myosins [9,10], associated with greater shortening velocities and speeds of tension generation in the atria [8,11]. Protein isoforms controlling both passive [12–14] and active [15–17] force contributions vary between the heart chambers, often in a species-dependent manner. Despite the breadth of these comparisons, a systematic integration of such differences within a biophysical perspective, linking basic subcellular properties to tissue-level behavior, is lacking for humans.

The aim of the present study was therefore to compare left-atrial (LA – Table 1) and left-ventricular (LV) biomechanics in skinned human muscle fiber preparations at 37 °C. An important regulator of active muscle contraction in this context is tissue strain. This so-called length-dependent activation (LDA) [18] has been presented as the microscopic underpinning of the Frank-Starling effect [19–23]. Although LDA is arguably the cumulative outcome of multiple subcellular mechanisms acting in parallel, we recently identified thick-filament activation as a potentially dominant contributor [24]. Within this paradigm, LDA emerges as a consequence of the force-dependent transitioning of myosin heads from an inactive “OFF” state to an active “ON” state [25–29]. We are not aware of a systematic quantitative comparison of LDA in the atria and ventricles. We therefore designed the present study to achieve this within the OFF/ON paradigm.

This study involved, firstly, performing comprehensive experimental force measurements on LA and LV muscle fiber preparations, encompassing both isometric steady-state and dynamic conditions, with a particular emphasis on resolving LDA properties. Secondly, we used these experimental measurements to calibrate computational biomechanical models for the LA and LV separately. Our Bayesian statistical methodology allowed not only a determination of the “most likely” parameter sets for each model, but also an assessment of LA/LV parameter differences [30]. These models hence provide a platform for exploring physiological implications in terms of chamber-specific LDA behavior and the energetic costs of contraction.

To our knowledge, this study is the first to systematically compare human LA and LV cardiomyocyte mechanics by directly linking sarcomere-level mechanisms to tissue-level force generation. This comparative characterization of cellular mechanical behaviors is an important step toward elucidating their functional roles at the whole-heart level. Ultimately, achieving a thorough and detailed understanding of cardiac function opens avenues for further development, not least in the perspective of identifying mechanisms of heart failure.

## 2. Methods

### 2.1. Experimental measurements

Tissue samples (in total 67 for LV, 59 for LA, derived from 9 organ donors; details in Supplementary Tables S1 and S2) were procured after informed consent from a legally authorized representative. Details of the procurement system have been published [31]. Procedures were approved by the University of Kentucky Institutional Review Board (#46103).

**Fig. 1.**
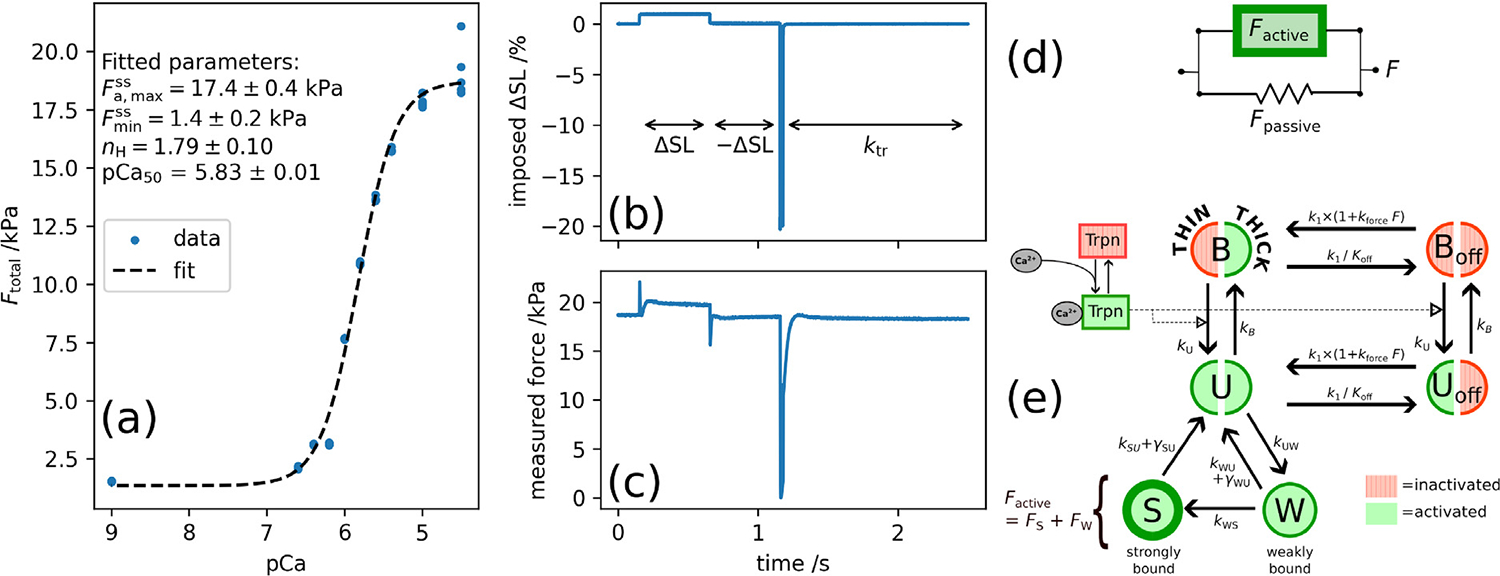
Outline of experimental measurements and analysis methods. (a) Representative force-calcium relationship data and fit to the Hill function (Eq. (1)). (b) Representative trace of the imposed changes in sarcomere length, starting from the isometric steady state, featuring a step change ±1%, a reversed step change ∓1%, and a tension recovery measurement after a 20-ms-long fiber slackening. (c) Representative force response to be analyzed as detailed in Fig. 3. (d) Overall theoretical model framework, consisting of a passive elastic element in parallel with the active contraction force. (e) Kinetic scheme for active force generation. Each state symbol represents the activation status of the thin (left shell) and thick filaments (right shell). In the B and U states, the thin filaments are, respectively, inactivated and activated by calcium-troponin, with the thick filaments in the “ON” state. $B_{\text{off}}$ and $U_{\text{off}}$ correspond to the inactivated thick filaments, the “OFF” state. Filament binding, represented by joining the shells, leads from U to the weakly bound state W. The myosin power stroke occurs in the transition from the weakly bound U state to the strongly bound S state. The ODE system is detailed in the Supplement.

Preparations were chemically permeabilized as described previously [32] and stretched to a sarcomere length (SL) of either 1.94±0.03μm or 2.23±0.03μm. (See Table S1 for sample allocations.) The choice of measuring a given preparation at a single length, also adopted in other studies [33,34], was found to be necessary to limit the introduction of systematic error arising from run down (i.e., drop in active force during the experiment). Tissue cross-sectional areas were estimated by video microscopy at (3.7 ± 2.2) × 10^−8^ m^2^. Preparations were activated in solutions with $\text{pCa}\;(=-{\text{log}}_{10}(\left[{\text{Ca}}^{2+}\right]/\text{mol}\;1^{-1}))$ values ranging from 9.0 to 4.5 (Fig. 1(a)). See Supplement for solution compositions.

Experiments were performed at 37 °C using SLControl software [35] and a commercially available force transducer (Model 403, Aurora Scientific, Ontario, Canada) and motor (Model 312, Aurora Scientific). SL was set in relaxing solution using video microscopy. It was not controlled during calcium activations because striations can be difficult to visualize in human myocardium during prolonged contractions at 37 °C, as also reported by other authors [36]. In our experience, preparations that exhibit substantial shortening during activations also exhibit greater run down of force. Run down was calculated as the percent difference between the maximum isometric tensions (pCa 4.5) measured at the beginning and end of the experiment. Preparations were excluded from the final analysis if they showed run down in excess of 30% or if they yielded a fitted force-pCa curve with $R^{2}<0.9$.

Once force reached steady state, small rapid step-length changes (ΔSL±1%, step-time ~ 0.5 ms) were imposed to assess transient kinetics (Fig. 1(b,c)). Next, the rate of tension recovery $\left(k_{\text{tr}}\right)$ was quantified using a rapid shortening/re-stretch protocol (20% muscle length, 20 ms duration).

### 2.2. Steady-state characterization (F-pCa)

For each experimental preparation, the isometric steady state was characterized by fitting a Hill curve to the force magnitude $F_{\text{total}}$ plotted as a function of pCa at fixed SL (see example in Fig. 1(a)):

(1) $$F_{\text{total}}(\text{pCa};\text{SL})=F_{\text{min}}^{\text{ss}}(\text{SL})+\frac{F_{\text{a},\text{max}}^{\text{ss}}(\text{SL})}{1+10^{n_{\text{H}}\left(\text{pCa}-{\text{pCa}}_{50}\right)}}$$

where $F_{\text{min}}^{\text{ss}}$ is the minimum steady-state force at low [Ca^2+^] (pCa = 9, associated with passive force generation); $F_{\text{a},\text{max}}^{\text{ss}}$ is the maximal steady-state Ca^2+^-associated force (associated with active contraction, pCa = 4.5); pCa_50_ measures the net calcium sensitivity; and the cooperativity index $n_{\text{H}}$ reflects the steepness of the force increase.

LDA was characterized to lowest order in terms of linear dependences on SL [28,37,38]. We used linear mixed-effects regression (LMER) analysis [33,34,39–43] to resolve the hierarchical LA/LV chamber dependence of LDA behavior in the presence of experimental noise and of random sample- and patient-wise variations (details in Supplement). This characterization returns mean values of the observables ($F_{\text{a},\text{max}},F_{\text{min}},n_{\text{H}}$, and pCa50) and their corresponding gradients (d/dSL), all evaluated at the midpoint SL, together with the associated uncertainties.

### 2.3. Dynamics characterization

Starting from the isometric steady state at fixed pCa = 4.5, small rapid stretches ΔSL of ±1% were imposed (Fig. 1(b)). This was followed 0.5 s later by a rapid change -ΔSL back to the original length. The measured response F(t) to each length change (Fig. 1(c)) was normalized to $\tilde{F}(t)$ so that $\tilde{F}(t)=0,1$, and −1 represent, respectively, the isometric steady states before the length change and after positive and negative length changes. This normalization ensures a maximal decoupling of the kinetic behavior from the steady-state properties to facilitate the subsequent model calibrations.

A principal component analysis [44–46] (details in Supplement) was performed via a singular value decomposition of the ensemble of all the $\tilde{F}(t)$ traces (amalgamating all the LA and LV data at pCa = 4.5) to identify the n=12 most significant principal component functions $\psi_{j}(t)(j\in [0,n])$ [47]. The coefficients $c_{j}$ therefore described 95% of the variance in the dataset relative to the overall mean $\left\langle \tilde{F}(t)\right\rangle$:

(2) $$\tilde{F}(t)=\langle \tilde{F}(t)\rangle +\sum_{j=1}^{n}c_{j}\times \psi_{j}(t)+\text{residue.}$$

### 2.4. Theoretical model framework

Our model framework (Fig. 1(d,e)) [24] is based on an earlier human ventricular model of Land et al. [37]. A defining feature of the model is its inclusion of the so-called cross-bridge OFF state, a structural “parked” configuration of the thick filaments that prevents myosin heads from interacting with the thin filaments [28,29,48–51]. Growing evidence suggests that transitions between this OFF state and the activated ON state is controlled by sarcomere force, thereby producing a positive feedback [25,26]. The potential fundamental regulatory role of this mechanism, within the context of the Frank-Starling effect, is increasingly recognized [27].

Each system state (circular symbol) in Fig. 1(e) describes the activation status of the thick and thin filaments, represented by the red or green semicircular shells. The thin filaments become activated via a configuration change of tropomyosin in the presence of troponincalcium, represented by the vertical transitions B → U and $B_{\text{off}}\to U_{\text{off}}$. OFF/ON thick-filament activation (horizontal transitions $B_{\text{off}}\to \text{B}$ and $U_{\text{off}}\to \text{U}$) is controlled by the force-dependent feedback mechanism [24]. Filament interactions (represented graphically by the joining of the shells) can occur only when both filaments are activated (state U). Cross-bridge binding leads from U to the weakly bound pre-stroke state W and hence, irreversibly, to the strongly bound post-stroke state S. The transition kinetics from W and S back into U contains components governed by their respective cross-bridge distortions $\zeta_{\text{S}}$ and $\zeta_{\text{W}}$, which follow a simple distortion-decay model (with rate constants $\gamma_{\text{WU}}$ and $\gamma_{\text{SU}}$) [37]. Mathematical details are provided in the Supplement.

Active force generation is expressed, following the Land model [37], as

(3) $$F_{\text{active}}=\frac{T_{\text{ref}}}{r_{\text{S}}}\left[\left(\zeta_{\text{S}}+1\right)S+\zeta_{\text{W}}W\right]$$

where $T_{\text{ref}}$ is a constant that sets the force magnitude scale, $r_{\text{S}}$ is a steady-state (“ss”) partial duty ratio for state S (defined as $r_{\text{S}}=\left.S_{\text{ss}}/\left(U_{\text{ss}}+W_{\text{ss}}+S_{\text{ss}}\right)\right)$. The present framework departs from the original Land model by removing all explicit length-dependent terms, namely the ad hoc parameters $\beta_{0}$ that scaled the peak tension with length and $\beta_{1}$ that modulated troponin’s [Ca^2+^] sensitivity [37]. Whereas LDA in the Land model relied on non-zero values of $\beta_{0}$ and $\beta_{1}$, length dependences in the force magnitude and calcium sensitivity here emerge naturally via the state populations S and W, which sense changes in SL indirectly via the force-dependent transition $k_{1}$. Thus, the ON/OFF balance is governed by the ratio of the forward $\left(k_{1}\right)$ to backward rate constants as $K_{\text{OFF}}\times \left(1+k_{\text{force}}F_{\text{total}}\right)$, where $K_{\text{OFF}}$ and $k_{\text{force}}$ are constants and $F_{\text{total}}=F_{\text{active}}+F_{\text{passive}}$ includes both active and passive (i.e., Ca^2+^-indendent) components. In the steady state,

(4) $$\frac{B}{B_{\text{off}}}=\frac{U}{U_{\text{off}}}=K_{\text{OFF}}\times \left(1+k_{\text{force}}F_{\text{total}}\right).$$

We assumed the passive force to obey linear-spring behavior

(5) $$F_{\text{passive}}(\text{SL})=a\left(\frac{\text{SL}}{{\text{SL}}_{0}}-1\right)$$

where SL_0_ is the effective resting length and a represents an effective stiffness. Although the passive mechanical behavior is well reported to be nonlinear [37,52,53], this linear formula provides the simplest approximation adapted to our experimental measurements, which were done at two SL values. Using simulations, we tested the potential impact of neglecting the nonlinearity on our overall analysis and estimated it to be relatively minor (see Supplement).

The 20 basic parameters defining the model and listed in Table 2 were constrained using the available experimental data. Following the original Land model, to facilitate calibration, the model is constructed such that none of the time-related parameters (i.e., with units of s^−1^) impact the steady-state properties. Kinetic rate constants outside the set of basic parameters (e.g., $k_{\text{WU}},k_{\text{SU}}$, and $k_{\text{B}}$) were calculated from the basic rate constants and the “partial duty ratios” $r_{\text{S}}$ and $r_{\text{W}}$. We assumed the values of $k_{\text{U}}$ and $k_{\text{TRPN}}$ quoted in the Land model [37] because they relate to the dynamic response of the system to a change in [Ca^2+^], which was not included in our experimental plan.

### 2.5. Model calibration general workflow

We calibrated representative LA and LV models by determining the respective parameter sets that were statistically most likely to predict the measurements. Details of the procedure are provided in the Supplement. In summary, a likelihood function ℒ was defined in the form of a multivariate Gaussian in terms of the mean values and uncertainties of each observable (Equation S29 in the Supplement). Preliminary parameter estimates were then obtained by first performing a Nelder–Meade optimization to determine the LDA-associated parameters ($K_{\text{OFF}},k_{\text{force}},\;a,{\text{SL}}_{0},{\text{pCa}}_{\text{T}50}^{\text{ref}},T_{\text{ref}})$. The remaining parameters were estimated by maximizing the likelihood function ℒ computed at 10^6^ points sampled over a Latin hypercube spanning three orders of magnitude. Starting from these initial estimates, we then performed a Markov Chain Monte Carlo analysis to estimate the posterior parameter distributions that map onto the observables’ distribution [54–56]. This process involved following the progression of 36 “walkers” exploring the 18-dimensional parameter space, guided by the likelihood function.

### 2.6. Tension redevelopment $\left(k_{\text{tr}}\right)$ simulations

Tension redevelopment experiments were simulated for SL=2.2μm. Starting from the isometric steady state, the model was made to contract under zero effective external load for 20 ms by adjusting SL to nullify the total force. SL was then restretched to its original value. The subsequent time progression of the force was then monitored as the system returned to the steady state.

For a given simulated trace, the rate of tension redevelopment was quantified as $k_{\text{tr}}=\text{ln}\;2/t_{1/2}$, where $t_{1/2}$ is the time required for the force to recover monotonically halfway from its minimum value after the restretch to the steady-state force.

## 3. Results

### 3.1. Steady-state characterization

**Fig. 2.**
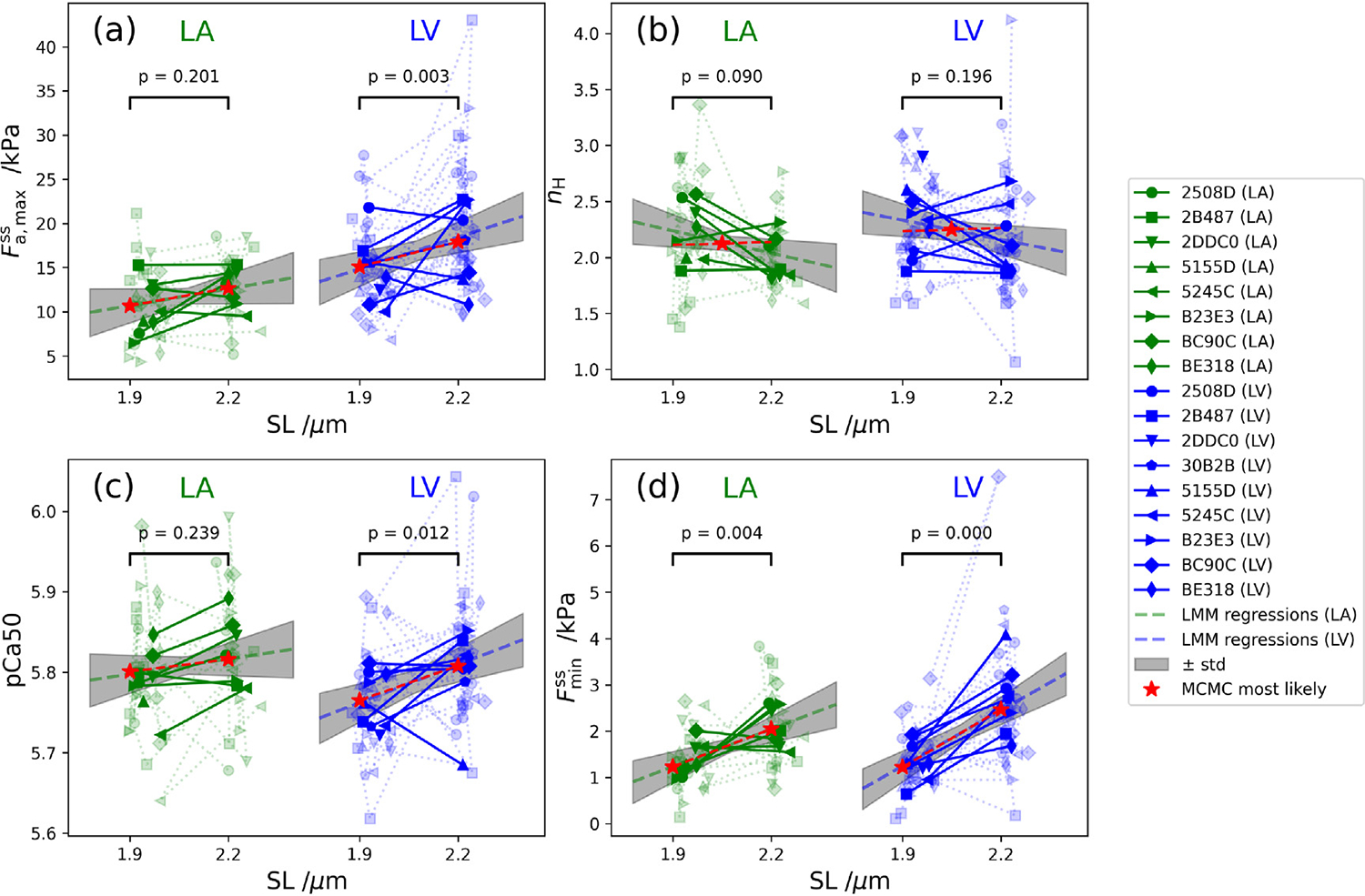
Length dependences of steady-state FpCa features. Experimental measurements of (a) $F_{\text{a},\text{max}}^{\text{ss}}$, (b) $n_{\text{H}}$, (c) pCa50, and (d) $F_{\text{min}}^{\text{ss}}$ are plotted as functions of SL for LA and LV preparations. Light-colored symbols denote measurements on individual muscle preparations for each patient (patient IDs listed in legend). Dark-colored symbols represent the patient-wise averages. [N.B. The “lone point” (LA upward-pointing triangle) in each subplot identifies the last remaining LA sample available from patient 5155D (see Table S1). Although a more complete and balanced sample set would have been preferable, linear mixed-effects regression (LMER) analysis is equipped to accommodate such gaps in sparse datasets in a statistically sound manner.] The dashed lines represent the LMER fit, and the gray shaded regions indicate the uncertainty bounds in the slope and vertical offset. The p-values express the degree of significance in the fixed-effect discrepancies. The red symbols denote the predicted values of the final calibrated models.

$F_{\text{a},\text{max}}^{\text{ss}},F_{\text{min}}^{\text{ss}}$, pCa_50_, and $n_{\text{H}}$ (Eq. (1)) were extracted from the LA and LV datasets at the two set SL values 1.9 and 2.2μm. Results are plotted in Fig. 2 as the green and blue data points, with each symbol representing one patient. (A representative dataset used for fitting is shown in Fig. 1a.)

The large random spread in the measurements is a typical feature of such measurements, as commonly reported in the literature [33,39–41]. Furthermore, the data points cannot be considered as independent measurements because of the uneven distribution of the measurements with respect to the human donors. The purpose of the LMER analysis was therefore to resolve the systematic underlying trends in the data while taking into account the inherent patient-based hierarchical structure of the datasets. Each regression calculation yielded the central value of the quantity, evaluated at the midpoint ${\text{SL}}_{\text{c}}=(1.9\;\mu \text{m}+2.2\;\mu \text{m})/2=2.05\;\mu \text{m}$, as well as the gradient with respect to SL. The resulting linear relationships are displayed in Fig. 2 (dashed lines) together with the associated uncertainties (shaded regions).

The signature features of LDA are clearly apparent in Fig. 2, namely an increase in both $F_{\text{a},\text{max}}^{\text{ss}}$ and pCa_50_ with increasing SL. However, these effects appear to be considerably weaker for LA than $\text{LV}\left(dF_{\text{a},\text{max}}^{\text{ss}}/d\text{SL}=\right.7.2\pm 6.3\;\text{kPA}/\mu \text{m}$ for LA vs. 12.0±4.9kPa/μm for LV, and $d{\text{pCa}}_{50}/d\text{SL}=0.061\pm 0.077\;\mu {\text{m}}^{-1}$ for LA vs. $0.158\pm 0.060\;\mu {\text{m}}^{-1}$ for LV). (An alternative LMER fitting, treating the short and long SL values as a categorical variable, resolves the average values and uncertainties for each measured SL separately; see results in the Supplement.)

Both LA and LV show a significant increase in the passive force $\left(F_{\text{min}}^{\text{ss}}\right)$ with SL but less steeply for LA, suggesting a lower passive stiffness. For both chambers, $n_{\text{H}}$ decreases weakly with SL, albeit with a low degree of certainty (high p value).

The LMER fit results, summarized in Table 3(a), were used to define target values $\mu_{i}$ in the likelihood function, as described below.

### 3.2. Dynamics characterization

**Fig. 3.**
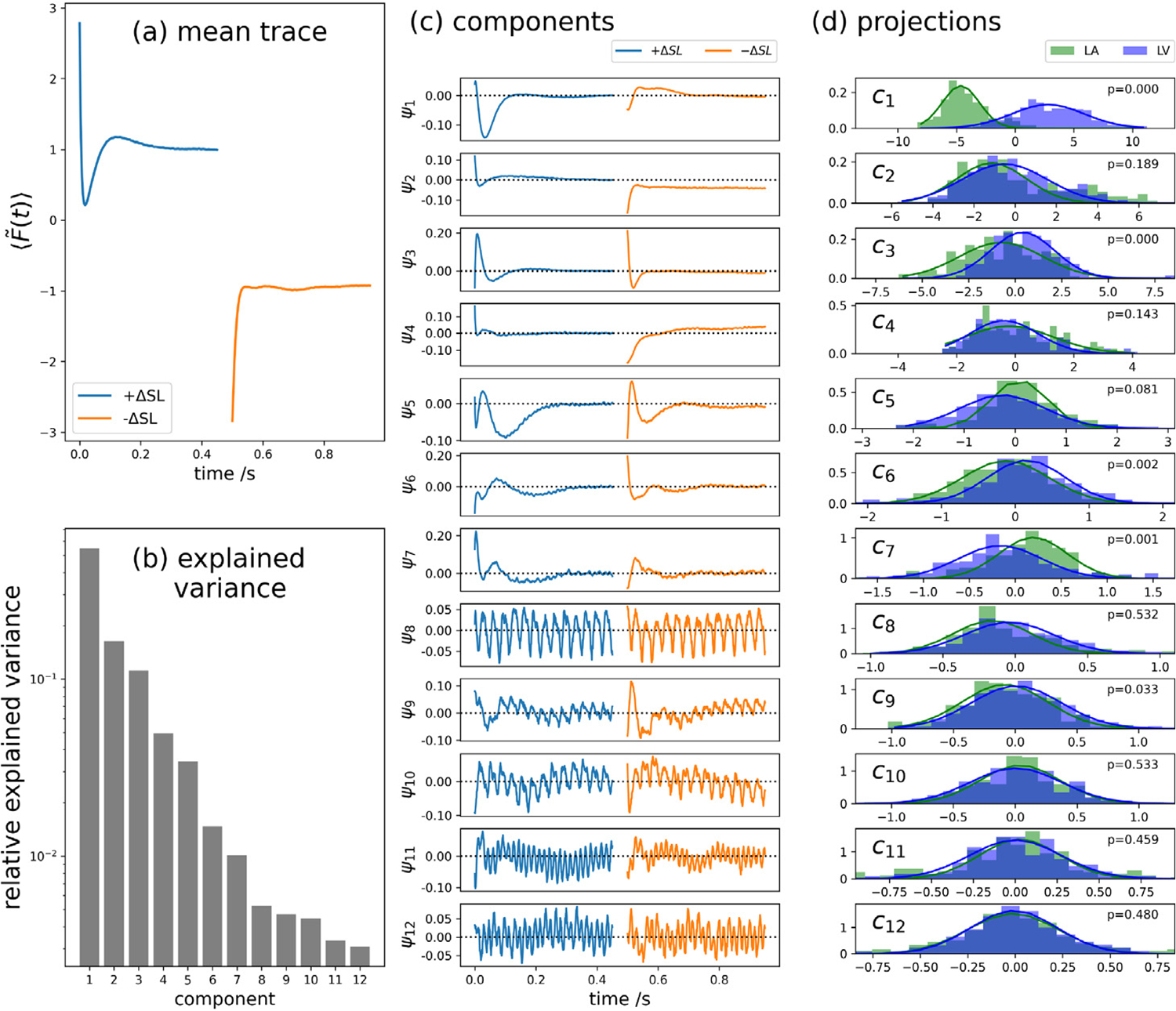
Principal component analysis of the quick-stretch length-step data traces. (a) Mean normalized trace $\left\langle \tilde{F}(t)\right\rangle$ showing the distinctive biphasic/monophasic asymmetry. (b) Proportion of explained variance for the corresponding $\psi_{j}$. The cumulative explained variance portion is 95% for j≤12 and 93% for j≤7. (c) Principal components $\psi_{j}(t)$ ordered according to their significance. (d) Distributions of the coefficients $c_{j}$ of the individual experimental quick-step responses projected onto the corresponding principal component basis set $\left\{\psi_{j}(t)\right\}$ (see Eq. (2)). The p-values are those of a two-sided t-test for equal mean values.

Positive/negative stretches produce a large instantaneous response, commonly associated with an immediate change in cross-bridge strain, which then decays toward a new steady state ($\tilde{F}\to \pm 1$ in Fig. 3(a)). The response is typically asymmetric, displaying a multiphasic response for ΔSL>0 and a rapid monophasic response for ΔSL<0, consistent with previously reported experiments [37]. Although for clarity two colors are used in Fig. 3 to differentiate between the positive and negative stretches, we combined both halves to form each $\tilde{F}(t)$ data trace to preserve the asymmetry within the principal component analysis.

The first n=12 principal components $\left(\psi_{j}(t),j\in [1,n]\right)$, plotted in Fig. 3(c) in order of decreasing significance, account for 95% of the total variance (Fig. 3(b)). The substantial increase in noise for j≥8 signifies that, beyond this limit, variations in the data traces are effectively indistinguishable from measurement noise. We therefore retained only the seven “useful” principal components, j∈[1,7], for all further analysis, which captured 93% of the overall variance.

The $c_{j}$ distributions obtained for LA and LV are compared in Fig. 3(d). The separation of the $c_{1}$ distributions, associated with the most significant principal component, together with the near-total overlap in all the distributions, confirms the existence of a systematic intrinsic difference between the dynamic behaviors of LA and LV.

**Fig. 4.**
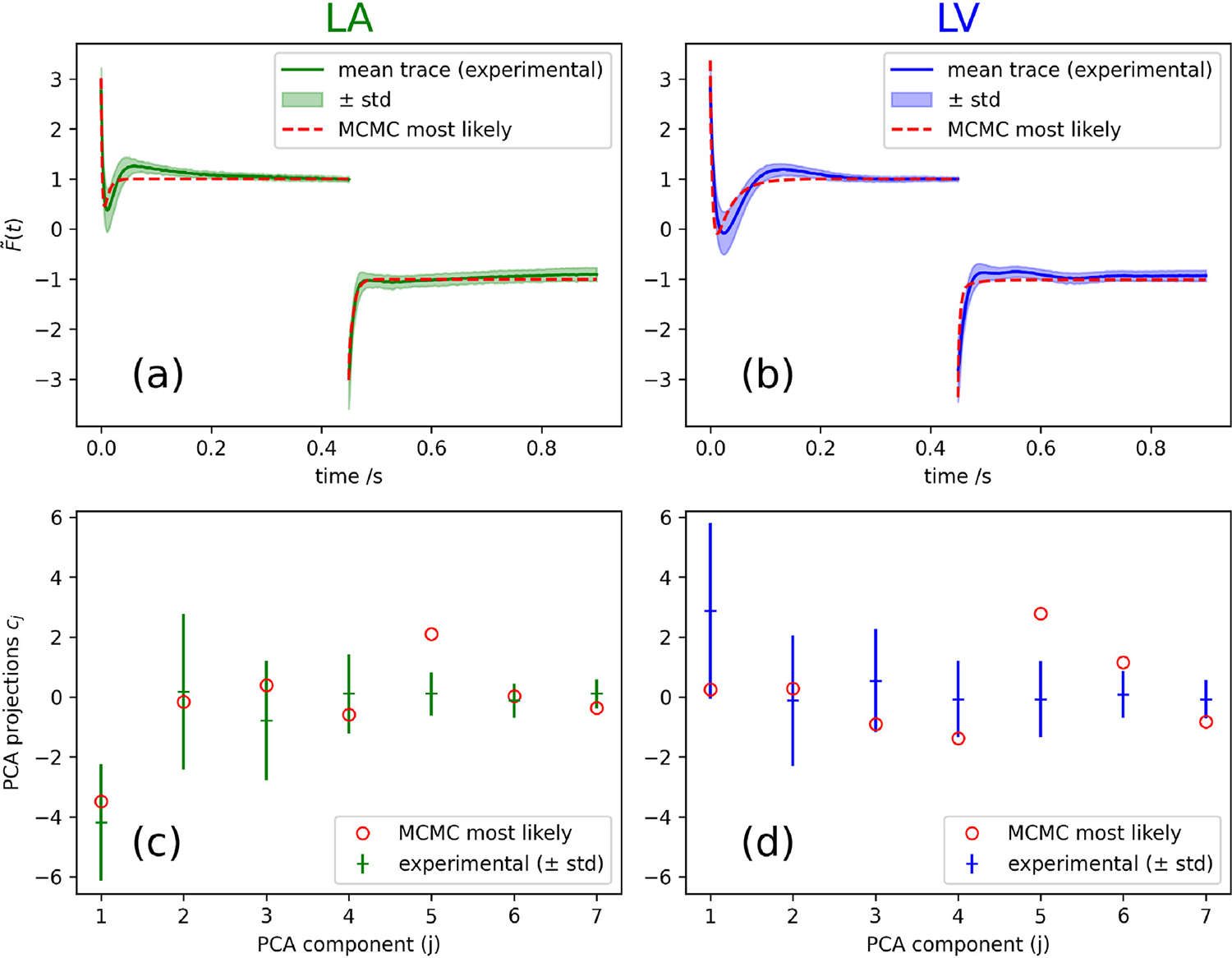
Quick-stretch force response. Mean traces for the normalized force $\tilde{F}(t)$ are plotted as solid curves for the (a) LA and (b) LV experimental data. Shaded regions indicate the standard deviations around the mean. Distributions of the corresponding principal-component projections $c_{j},j\in [1,7]$ are represented, respectively in (c) and (d), indicating the mean values and standard deviations of the distributions shown in Fig. 3(d). The red symbols represent the $c_{j}$ values predicted by the MCMC-calibrated models. The corresponding $\tilde{F}(t)$ curves are plotted as the red dashed curves in (a) and (b).

The mean normalized quick-stretch force traces $\tilde{F}(t)$ for LA (green curves) and LV (blue) are compared in Fig. 4(a,b), after discarding the noise-dominated components $c_{8}-c_{12}$.

The negative overshoot, clearly displayed in both the LA and LV data following the positive stretch (first half of the curve), is more pronounced for LV than LA. This effect is the manifestation, in temporal space, of the relative signs of the average $c_{1}$ values (topmost plot in Fig. 3(d)), which are associated, in the principal-component space, with the strong negative dip in $\psi_{1}(t)$ (topmost plot in Fig. 3(c)).

Taken together, the means and standard deviations of $c_{j}$, displayed in Fig. 4(c,d) and listed in Table 3, define the target values $\mu_{i}$ describing the dynamic behavior for the purpose of the model calibrations.

### 3.3. Model calibrations

**Fig. 5.**
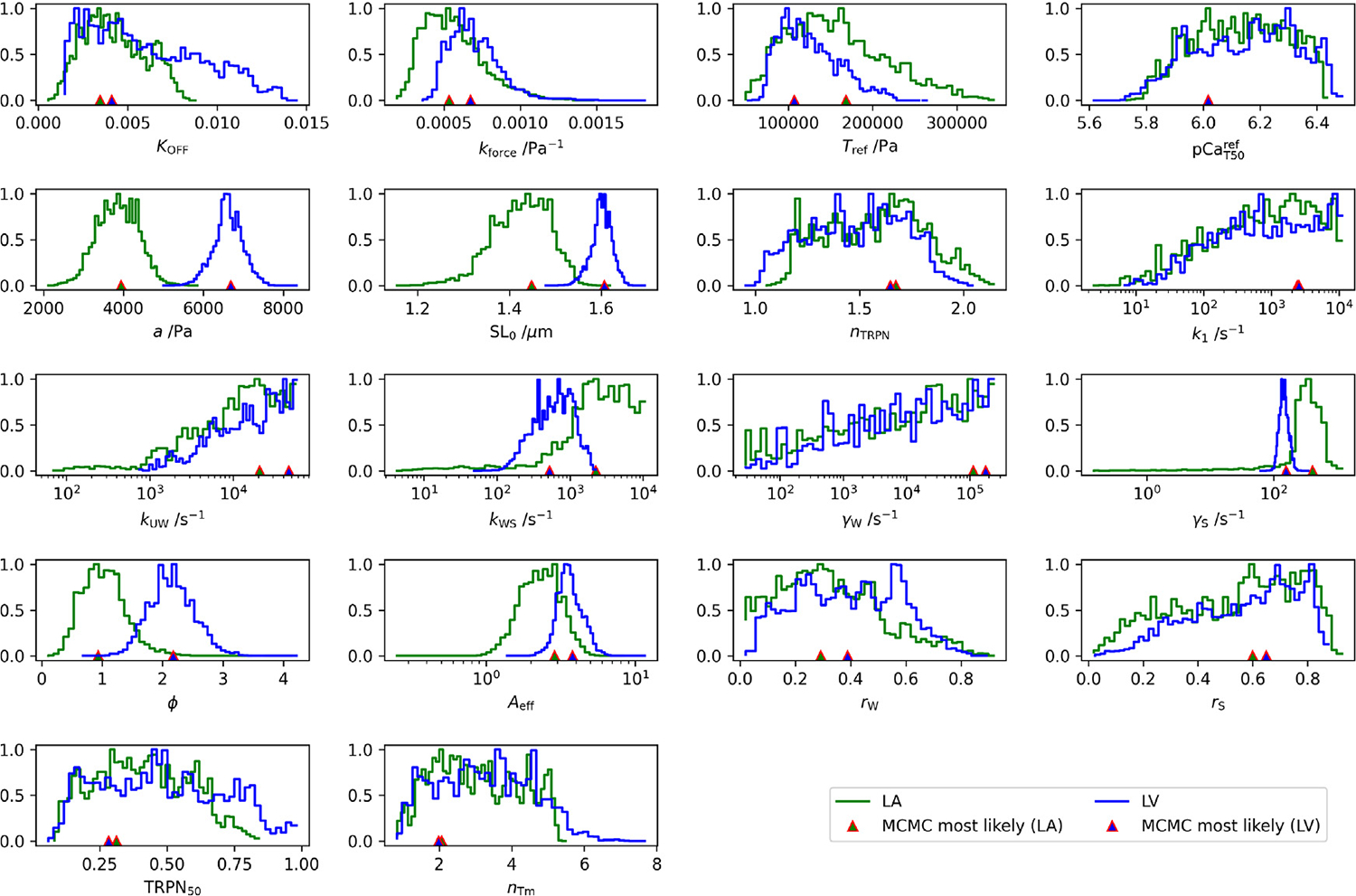
MCMC-determined likelihood distributions of the basic model parameters. Maximum likelihood distributions ℒ[θ] (normalized to their maximum value) are plotted for each model parameter (contained in the parameter set θ) and for each region LA and LV, as determined by the MCMC analysis (6 × 10^5^ simulation steps, 36 walkers; details in Supplement). The maximum-likelihood locations are indicated by the triangular symbols on the horizontal axis.

The Bayesian calibration method mapped the experimental observables (Table 3) onto model parameter distributions. This was done by optimizing the likelihood values computed from the MCMC walker trajectories (see Supplement) over > 3 × 10^5^ steps. The resulting posterior distributions are plotted in Fig. 5, normalized by their maximum value to facilitate LA/LV comparisons.

These distributions serve two purposes. Firstly, they indicate the extent to which each parameter is constrained by the experimental data. For example, a and SL_0_ are well constrained, as expected from the clear linear dependence of $F_{\text{min}}^{\text{ss}}$ versus SL visible in Fig. 2(d). In contrast, the $k_{1},k_{\text{UW}}$, and $\gamma_{\text{W}}$ distributions cover one or more orders of magnitude, indicating a low degree of constraint by the available data. For each chamber LA and LV, the parameter set $\theta_{\text{best}}$ (associated with the maximum likelihood value $\left.ℒ\left[\theta_{\text{best}}\right]\right)$ defines the model most consistent with the experimental dataset for that chamber. The protocol used to construct the distributions in Fig. 5 (see Methods) makes the individual parameters of this optimal set identifiable from the distribution maxima.

Secondly, the degree of separation of the LA and LV distributions in Fig. 5 reflects chamber-specific differences in the parameters. Thus, the clear separations of the a, SL_0_, $\gamma_{\text{S}}$, and ϕ distributions indicate unambiguous regional dependences. At the other extreme, the strong overlaps in the $K_{\text{OFF}},{\text{pCa}}_{\text{T}50}^{\text{ref}},n_{\text{TRPN}},r_{\text{W}},r_{\text{S}},$ TRPN_50_, and $n_{\text{Tm}}$ distributions imply that LA/LV difference for these parameters are not resolvable using the present data.

To assess the calibration quality, the model predictions are superposed onto the corresponding experimental results in Figs. 2 and 4. For both LA and LV, the simulations (red symbols in Fig. 2) accurately predict the mean F-pCa results except for $dn_{\text{H}}/d\text{SL}$, which, as explained above, was not included in the likelihood calculations. Nonetheless, the predicted gradients $dn_{\text{H}}/d\text{SL}\approx 0$ fall within the experimental error ranges. The predicted projections $c_{j}(j\in [1,7])$ of the dynamic responses, plotted as the red symbols in Figs. 4(c, d), generally fall within the error range defined by the mean values and standard deviations, except, intriguingly, for component j=5. This component accounts for only approximately 3% of the overall data variance (Fig. 3(b)) and is therefore unlikely to impact the overall fit quality significantly. We evaluate this quality by plotting the predicted quick-stretch traces in the time domain as the red dashed curves in Figs. 4(a, b). The results are reasonably consistent with the mean experimental curves and their associated error range.

### 3.4. Model validations

We used the tension redevelopment $\left(k_{\text{tr}}\right)$ measurements, performed at fixed SL=2.2μm, to validate the calibrated chamber-specific models. Although these measurements were made in conjunction with the quick-stretch measurements, they were not included in the parameter calibration process, in order to constitute an independent validation dataset. No further parameter constraining was applied when comparing the $k_{\text{tr}}$ experimental and simulation results.

**Fig. 6.**
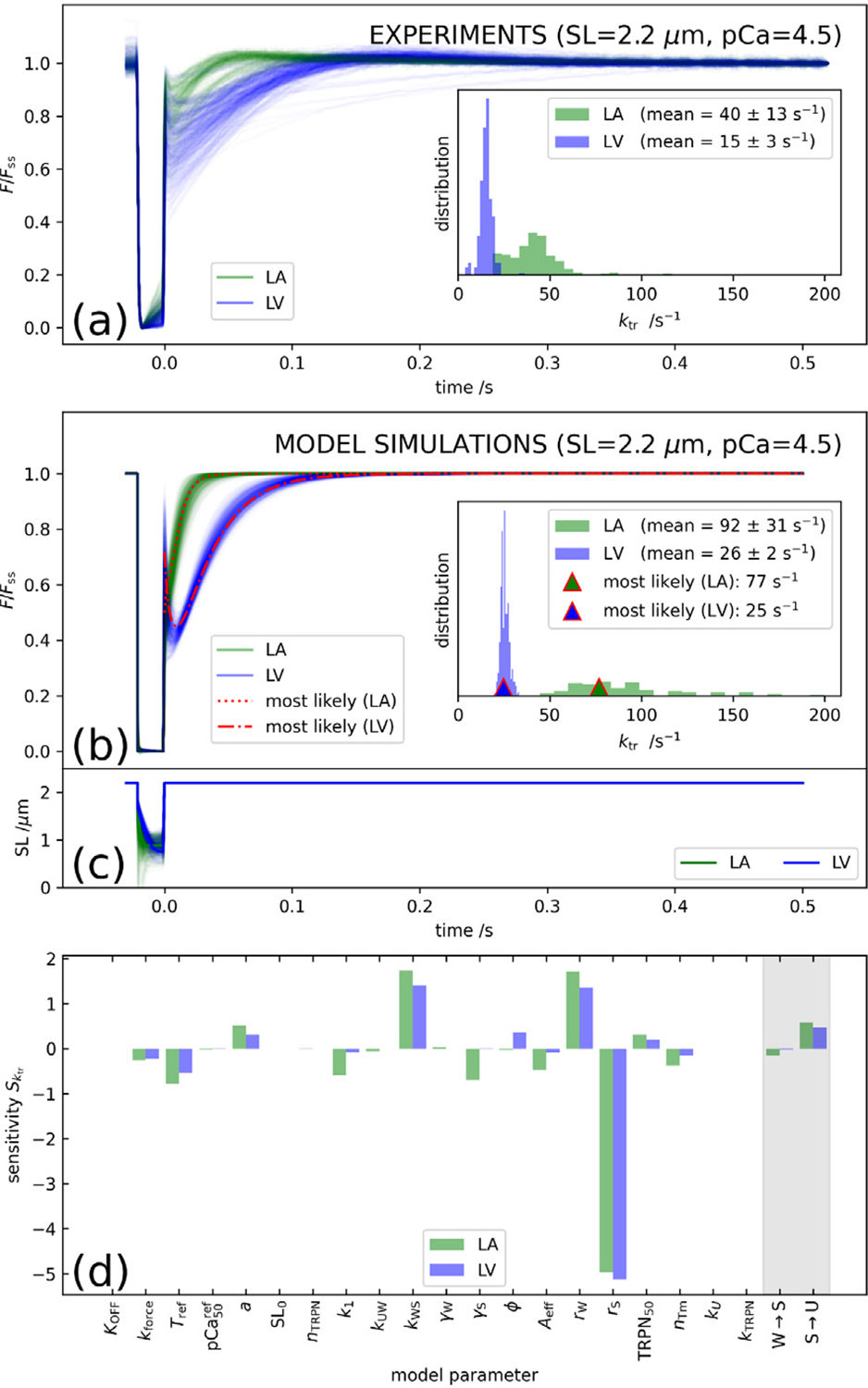
Experimental and simulated tension redevelopment $\left(k_{\text{tr}}\right)$ experiments. (a) Normalized experimental measured traces of the $k_{\text{tr}}$ protocol (slack period = 20 ms) with recovery to SL=2.2μm) are plotted for the LA and LV data. The inset shows the distribution of $k_{\text{tr}}$ values, calculated from the data traces as $k_{\text{tr}}=\text{ln}\;2/t_{1/2}$, where $t_{1/2}$ is the time needed for the force to recover halfway from its minimum (“residual”) value after restretch to the steady state force. Average normalized residuals $F_{\text{residual}}$ are 0.77 ± 0.06 for LA and 0.64 ± 0.05 for LV (t-test p<0.001). (b) Analogous simulated $k_{\text{tr}}$ results, predicted using 500 models randomly sampled from the MCMC output. The red traces and red triangles in the inset represent the model results returned by the MCMC analysis. Average normalized residuals $F_{\text{residual}}$ are 0.46 ± 0.09 for LA and 0.42 ± 0.04 for LV (t-test p<0.001).(c) The SL time courses imposed on the sampled models to implement the zero-load isotonic contraction in the slackening phase. (d) Bar chart showing the sensitivities of the LA and LV models to each basic model parameter p, defined as $S_{k_{\text{tr}}}[p]=\partial k_{\text{tr}}/\partial p\times p/k_{\text{tr}}$ (see Supplement for details). The bars in the shaded region represent the sensitivities with respect to the transitions W→S and S→U in isolation, calculated by effectively varying the *derived* parameters $k_{\text{WS}}$ and $k_{\text{SU}}=k_{\text{WS}}r_{\text{W}}\left(1/r_{\text{S}}-1\right)$ while adjusting $r_{\text{W}}$ and $r_{\text{S}}$ so as to fix all but one transition rate constant at a time.

The measured force traces, normalized to their final steady-state value, are plotted in Fig. 6(a). Upon completion of the restretch phase (time = 0), the traces typically show a sudden rise, commonly associated with the instantaneous stretching of bound cross bridges. A rapid decrease in force follows, associated with force-induced cross-bridge detachment, reaching a minimum “residual” value. ($F_{\text{residual}}^{\text{LA},1.9\;\mu \text{m}}=0.76\pm 0.07,F_{\text{residual}}^{\text{LA},2.2\;\mu \text{m}}=0.77\pm 0.06,F_{\text{residual}}^{\text{LV},1.9\;\mu \text{m}}=0.56\pm 0.05,F_{\text{residual}}^{\text{LV},2.2\;\mu \text{m}}=0.64\pm 0.05$. T-test comparisons give p-values < 0.005 for all pairs except for LA 1.9vs2.2μm(p=0.7).) The subsequent gradual recovery toward the isometric steady state is quantified by an effective time constant $k_{\text{tr}}$. The distributions of $k_{\text{tr}}$ values are plotted in the inset. Tension recovery is faster for LA (mean $k_{\text{tr}}=40\pm 13{\;\text{s}}^{-1}$) than LV (15 ± 3 s^−1^) by a factor of 2 ~ 3, consistent with the noticeably faster dynamics exhibited in the LA quick-stretch measurements, compared to LV, apparent in Figs. 4(a, b).

Fig. 6(b) shows the corresponding simulated results. To quantify the variation in the predictions, we randomly sampled 500 models from the MCMC output and plotted the corresponding predicted time courses (green and blue curves). Corresponding $k_{\text{tr}}$ distributions, determined in the same manner as for the experimental curves, are shown in the inset (mean values 83 ± 23 s^−1^ for LA, 25 ± 2 s^−1^ for LV). The calibrated LA and LV model predictions are plotted as red curves, consistent with the distribution means. We estimate the $k_{\text{tr}}$ values for the calibrated models as 77 ± 23 s^−1^ for LA and 25 ± 2 s^−1^. Although these values overestimate the experimental results by a factor of ~ 2, there is substantial overlap between the broad experimental and predicted distributions. We discuss potential causes for the discrepancies below. Nonetheless, the simulations reproduce the approximate 3-fold LA/LV difference observed experimentally.

We investigated the underlying cause of this LA/LV difference by calculating the relative sensitivity $S_{k_{\text{tr}}}$ of $k_{\text{tr}}$ with respect to each model parameter. Details of the calculation are provided in the Supplement. The bar charts in Fig. 6(d) identify $k_{\text{WS}}$ as the basic kinetic parameter (with units of s^−1^) to which $k_{\text{tr}}$ is most sensitive. Within the model framework, this parameter controls both the rate constants for the transitions W→S and S→U (see derived parameters in Table 2). To discriminate between these two transitions, we calculated their respective $S_{k_{\text{tr}}}$ values as derived in the Supplement. From the bars in the shaded region in Fig. 6(d), we find that $k_{\text{tr}}$ is significantly more sensitive to S→U than W→S, consistently for both the LA and LV models. This therefore identifies S→U as the transition that most strongly controls the tension redevelopment rate.

### 3.5. Comparing passive and non-passive contributions to LDA between LA and LV

We used the LA and LV models to investigate the mechanistic reasons for the stronger LDA behavior observed experimentally in LV (see Fig. 2). Because the models assume that LDA arises exclusively from the force-sensitive balance between the thick-filament OFF/ON states [24], this balance is potentially controlled by two classes of influence.

The first contribution is the intrinsic force sensitivity of the OFF/ON state balance in the thick filaments, which is governed by $K_{\text{OFF}}$ and $k_{\text{force}}$. A low $K_{\text{OFF}}$ pushes the balance of unbound myosins toward the OFF state, which increases the pool of recruitable cross bridges. A high $k_{\text{force}}$ implies a stronger force feedback to release myosins from the OFF state. As shown in Fig. 5, the ~30% shift in the LV $k_{\text{force}}$ distribution, compared to LA, favors enhanced LDA properties for LV, consistent with the experimental results.

The second source of influence is the passive force. We previously demonstrated that the passive mechanical properties plays a prominent role in enabling LDA. This provides the initial length-dependent signal needed to seed the OFF-state positive feedback (see Eq. (4)) [24]. The LV passive stiffness a clearly exceeds that of LA (Fig. 5), which therefore also favors stronger LDA for LV.

**Fig. 7.**
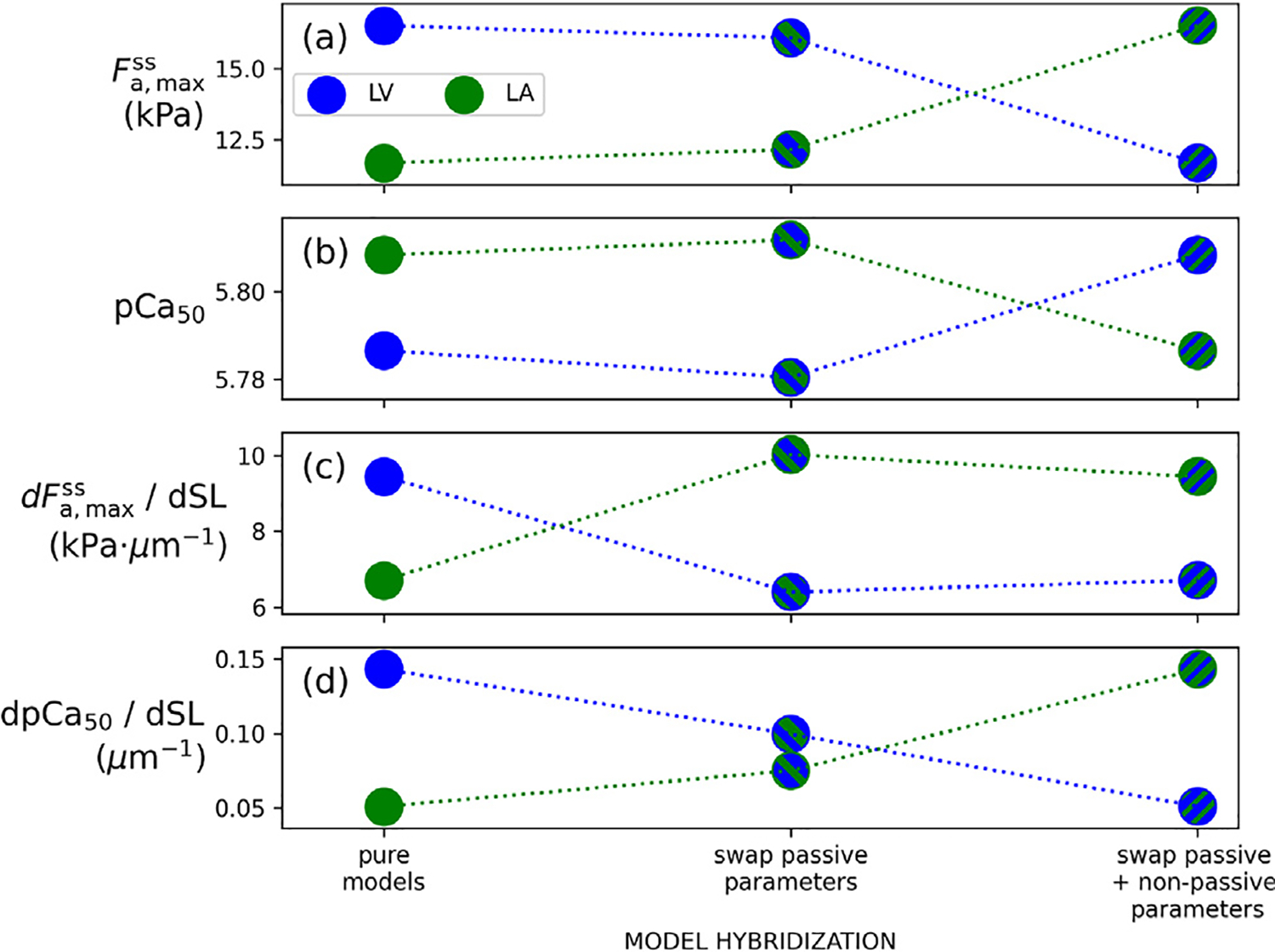
Comparing passive/non-passive contributions to LDA. Predictions of the maximum active force $\left(F_{\text{a},\text{max}}^{\text{ss}}\right)$, calcium sensitivity (pCa_50_) and their gradients (d/dSL) are shown for the calibrated LA and LV models as well as for hybrid models, constructed by substituting, in turn, the passive mechanical model parameters (a and SL_0_) and non-passive parameters (i.e., all remainin model parameters) taken from LA and LV. All quantities were evaluated at ${\text{SL}}_{\text{c}}=2.05\;\mu \text{m}$.

To compare the contributions of these two influences, we generated hypothetical hybrid models where we first permuted the LA and LV values of the passive model parameters (a and SL_0_) and then those of the non-passive (all remaining) parameters. The impact on the observables was monitored at each step. The predictions of $F_{\text{a},\text{max}}^{\text{ss}}$, pCa_50_, and their SL gradients (all evaluated at the ${\text{SL}}_{\text{c}}=2.05\;\mu \text{m}$) are displayed in Fig. 7. They show that the LA/LV differences in $F_{\text{a},\text{max}}^{\text{ss}}$ and pCa_50_ (Fig. 7(a,b)) are negligibly affected by permuting the passive parameters only, which suggests that these quantities are mostly determined by the cross-bridge properties instead. In contrast, permuting the passive parameters has a strong impact on the gradients $dF_{\text{a},\text{max}}^{\text{ss}}/d\text{SL}$ (Fig. 7(c)), with negligible impact from the other parameters, suggesting that this gradient is predominantly controlled by the passive mechanical properties. Permuting the passive parameters produces intermediary changes in $d{\text{pCa}}_{50}/d\text{SL}$ (Fig. 7(d)), which suggests that, for this LDA property, both passive and non-passive sarcomere properties contribute comparable amounts.

### 3.6. Comparing the energy costs of isometric force between LA and LV

Narolska et al. defined the energetic cost of force generation as the rate of ATP consumption per volume of preparation per amount of force produced. This effectively provides a measure of “muscle economy” [6,57]. They observed an approximate five-fold difference in this metric between skinned LA and LV strips at 20 °C (11.4 ± 1.4 vs. 2.4 ± 0.3 mmol kN^−1^ m^−1^ s^−1^, i.e., differing by a factor 4.8 ± 0.8), which suggests that, for a given amount of force generated, “the economy of force maintenance in ventricular tissue is about five times greater than in atrial tissue”. Within our modeling framework, an analogous force cost metric for isometric force under saturating [Ca^2+^] is given by

(6) $$C=S_{\text{ss}}k_{\text{SU}}/F_{\text{a},\text{max}}^{\text{ss}}.$$

This quantity represents the average cycling rate (with concomitant hydrolysis of one ATP) per myosin per amount of generated force. $S_{\text{ss}}$ denotes the steady-state occupancy of state S and $F_{\text{a},\text{max}}^{\text{ss}}$ the corresponding maximum active force. Using our calibrated models at fixed ${\text{SL}}_{\text{c}}=2.05\;\mu \text{m}$, we calculated C=1.57 and 0.67 s^−1^ kPa^−1^ for LA and LV, respectively. The ratio 2.34 is in qualitative agreement with Narolska’s results, reflecting a greater efficiency in LV to produce a given amount of force compared to LA.

We validate these simulation predictions more explicitly against the Narolska measurements using an order-of-magnitude calculation as follows. Assuming that a sarcomere contains ~ 300 myosins [58] and has an aspect ratio of 1 : 7 [59] (i.e., length ~2μm and width ~0.3μm), the average volume density of myosins is $\sim 3\;\text{mmol}\;{\text{m}}^{-3}$. Multiplying this value by our “per myosin” estimates for C (Eq. (6)) gives ~ 5 and ~2 mmol kN^−1^ m^−1^ s^−1^, for LA and LV, respectively, similar to Narolska et al.’s results.

The value of the energy cost thus hinges primarily on two quantities: $S_{\text{ss}}$ and $k_{\text{SU}}$. To identify which of these most significantly accounts for the LA/LV difference, we generated hybrid models where, starting from each calibrated model, we sequentially substituted the $S_{\text{ss}}$ and $k_{\text{SU}}$ values from the other model, while monitoring the corresponding impact on C. (See Supplement for details.)

**Fig. 8.**
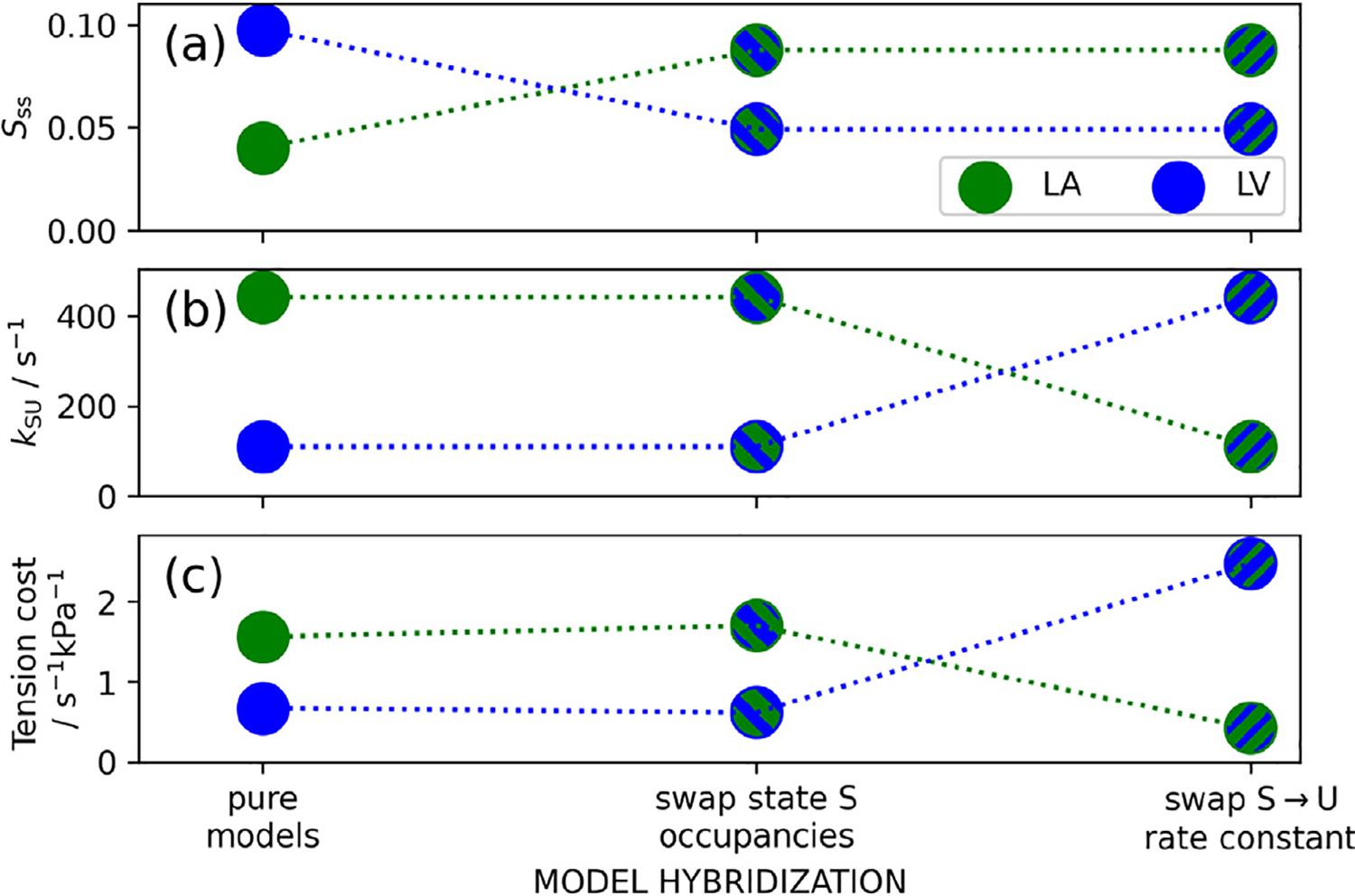
Comparing the contributions to the energetic cost of isometric force. We investigated which contribution more significantly to the force cost (Eq. (6)): the occupancy $S_{\text{ss}}$ of the strongly bound crossbridge state, or the rate constant $k_{\text{SU}}$ of crossbridge detachment. Starting from the calibrated models, hybrid models were generated, sequentially substituting (a) $S_{\text{ss}}$, (b) $k_{\text{SU}}$ while monitoring (c) the resulting force cost $C=S_{\text{ss}}k_{\text{SU}}/F_{\text{a},\text{max}}^{\text{ss}}$.

The results, displayed in Fig. 8(c), show that, whereas swapping $S_{\text{ss}}$ has a negligible effect on the force cost, swapping $k_{\text{SU}}$ values produces the approximate recovery of the initial values of the two models reciprocally. This result therefore identifies $k_{\text{SU}}$, rather than the occupancy of state S, as determining the difference in force cost.

## 4. Discussion

The aim of this study was, firstly, to compare experimental measurements of force generation dynamics between human LA and LV muscle fiber preparations at 37 °C; and, secondly, to elucidate the mechanistic origins of their differences using calibrated chamber-specific biomechanical models. Our Bayesian methodology aimed to minimize subjective bias in the model calibrations and to assess the statistical significance of individual parameter differences between the chambers.

The first important result is the systematic experimental characterization of the LA and LV mechanics, confirming clear differences between LA and LV biomechanics. A first observation is the larger active force $F_{\text{a},\text{max}}^{\text{ss}}$ (by ~ 50%) in LV than LA (Table 3). This compares with the approximate two-fold difference reported by Van der Velden et al. [36]. However, whereas they also reported a significantly lower calcium sensitivity in atria (by 0.13 pCa units), we did not observe this in pCa_50_. A second observation is that LV displays more pronounced LDA features than LA, as expressed in the SL dependences of both $F_{\text{a},\text{max}}^{\text{ss}}$ and pCa_50_ (Figs. 2(a,c) and Table 3). Although in absolute terms the gradients $dF_{\text{a},\text{max}}^{\text{ss}}/d\text{SL}$ differ clearly between LA and LV, in relative terms they vary in similar proportion (~ 50%) with $F_{\text{a},\text{max}}^{\text{ss}}$. The results are nonetheless consistent with stronger LDA in LV than LA, insofar as both chambers operate at similar sarcomere lengths [60,61]. Clinical studies have indeed reported the existence of the Frank-Starling effect in the human LA [2,5,62], albeit more weakly than in LV [15,63]. The present study clarifies this effect quantitatively in both chambers at the sarcomere level. A third observation is the faster dynamic force response to length change in LA than LV. This is reflected in both the small-stretch (Fig. 4) and tension redevelopment measurements and $k_{\text{tr}}$ values (Fig. 6) and is consistent with earlier measurements [7,57]. The clear separation of the dominant principal component $c_{1}$, shown in Fig. 3(d), unambiguously distinguishes between the LA and LV dynamics.

The second important result concerns the importance of the passive mechanical properties in controlling LDA. We previously demonstrated the potentially central role of passive mechanical properties in bringing about LDA [24]. In essence, that mechanism relies on length-dependent passive force, quantified by $d\;F_{\text{min}}/d\text{SL}$, to provide an initial signal that becomes amplified by the OFF/ON force feedback. Although this principle by itself does not predict the net strength of the emergent effects, the results of Fig. 7 suggest that it could be instrumental in controlling the strength of LDA quantitatively. This is particularly manifested by the active force magnitude $F_{\text{a},\text{max}}^{\text{ss}}$, whose length dependence appears to be predominantly affected by the passive properties (Fig. 7(c)). Although previous studies identified the participation of passive force in regulating LDA and the Frank-Starling effect [27,64], the present study is, to our knowledge, the first to explicitly link LA/LV differences in LDA to differences in their respective passive mechanical properties.

These functional differences may originate from chamber-specific differences in the isoforms of key proteins. The sarcomere-spanning protein titin is the major determinant of passive stiffness in myocytes [13,65]. Of the two isoforms found in cardiac muscle, N2BA is larger and more compliant than N2B. In humans, the atria express higher levels of N2BA, compared to the ventricles, consistent with their lower passive stiffness [13,66,67]. Our experimental measurements (Fig. 2(d)) manifest these differences in the lower calibrated values of the passive stiffness parameter a in LA compared to LV (Fig. 2 and Table 2). As our analysis shows (Fig. 7), this has a repercussion on the length-dependent regulation of active contraction and hence the Frank-Starling effect.

The importance of titin in active force development in muscle systems is increasingly recognized [64,68]. A titin-based modification of the calcium sensitivity of active force has been reported in mouse cardiomyocytes [69]. Using rat experimental models expressing different titin isoforms, several authors observed a clear correlation between the titin stiffness and SL-dependent changes in passive force, maximum active force, and pCa_50_, as well as cross-bridge cycling and $k_{\text{tr}}$, consistent with our results [70,71]. Similarly, Methawasin et al. reported an attenuation of the Frank-Starling mechanism resulting from increased titin compliance [72]. X-ray diffraction studies on both cardiac [73] and skeletal muscle systems [74] have highlighted a direct connection between titin strain on the sarcomere thin- and thick-filament structures, in particular favoring the thick-filament ON state. Within this perspective, the present modeling study strengthens the growing understanding of titin as a mechanosensitive regulator of active muscle contraction acting via OFF/ON thick-filament dynamics [27].

Nevertheless, this regulative role of passive mechanics remains the subject of debate, with some authors presenting seemingly contradictory evidence. Park-Holohan et al. measured the force dependence of cross-bridge structural orientations (taken as an index of OFF/ON-state occupation) [75] in the absence and presence of actin tension (achieved by varying [Ca^2+^]). They concluded that cross-bridge orientations are significantly more sensitive to active than passive filament stress, hence disputing the role of passive stress in mediating the Frank-Starling mechanism. Similarly, by measuring X-ray diffraction in intact trabeculae under diastolic (low [Ca^2+^)]) conditions, Caremani et al. observed no significant structural dependence of the thick filaments associated with passive stress alone, arguing against OFF-state dynamics being controlled by passive mechanics [76]. Our present results do not necessarily contradict those experimental findings. Although our modeling framework assumes passive stress as a necessary ingredient for LDA, the quantitative features of OFF/ON dynamics are also controlled by active force via the OFF-state feedback mechanism. To illustrate this effect directly, we compared the impact of length change on ON cross bridges (estimated as $\text{ON}=1-B_{\text{off}}-U_{\text{off}}$) under low (pCa = 9.0) and high calcium (pCa = 4.5) (see Supplement). For both the LA and LV models, the gradient $d\text{ON}/dF_{\text{total}}$ is 2.6 times greater for pCa = 4.5 than 9.0, indicating a stronger contribution from active thick-filament force to OFF-state cross-bridge recruitment. It is therefore plausible that the contribution of passive force to cross-bridge recruitment becomes manifest, under some experimental circumstances, only after sufficient amplification by the positive feedback.

Other computational modeling studies have sought to characterize mechanosensing function in muscle quantitatively in terms of underlying biophysical processes. Campbell et al. showed that a force-dependent OFF/ON transition rate could better account for LDA properties and the Frank-Starling effect than a force-independent rate [77]. By monitoring OFF/ON transitions in single-myosin heads in Monte-Carlo simulations, Marcucci demonstrated the ability of a titin-based mechanosensing mechanism in the thick filaments to predict Frank-Starling length dependences [78–80]. Our present simulations consolidate this understanding by emphasizing the potentially dominant role of OFF/ON transitions in regulating the length dependence of active force.

We calibrated our models against small fast length changes and validated them (without further constraining the parameters) against unseen measurements of tension redevelopment following large stretches $\left(k_{\text{tr}}\right)$. We observe a clear enhancement of $k_{\text{tr}}$ values in LA compared to LV, as reported by other authors [57]. While the calibrated models quantitatively predicted this relative enhancement, the average modeled $k_{\text{tr}}$ values overestimate the experimental ones approximately two-fold. We suggest two possible explanations for this. Firstly, the large length changes involved in the $k_{\text{tr}}$ protocol may reflect additional molecular mechanisms not discernible for small stretches. Second, we chose to fit the model to the principal components, which provide a compact unbiased representation of the full transient, rather than to specific features of the data traces. This choice may influence the fitted parameters.

An additional outcome of the $k_{\text{tr}}$ analysis was the identification of S→U, associated with the irreversible detachment from the strongly bound force-generating state S, as the likely main determinant of $k_{\text{tr}}$ (Fig. 6(d)). Using an explicit cross-bridge model, Wang and Kawai [81] argued that $k_{\text{tr}}$ is limited by the smallest rate constant in the cycle, in contrast with early analyses that attributed $k_{\text{tr}}$ instead to the fastest rate constant [82,83]. Using an altogether different analytical approach, our present results shows consistency with Wang and Kawai’s prediction.

The observed $k_{\text{tr}}$ force residuals were greater for LA (0.77 ± 0.06) than LV (0.64 ± 0.05) (p<0.001), a trend reproduced qualitatively in the simulations (0.46 ± 0.09 vs. 0.42 ± 0.04, p<0.001). Systematic experimental measurements – and their interpretation – of these residuals are notoriously challenging. Quantitatively, their values depend on conditions that include the metabolites, muscle type and species, and even the millisecond profile of the applied length changes. Reported measurements span low (typically ≲ 0.25 [84–88]) to larger values (≳ 0.5 [89]). Piroddi et al. reported ~ 0.4 for LA and ~ 0.65 for LV [7], a trend opposite to our observations. However, we are not aware of sufficient other human-derived data at 37 °C that would allow a meaningful comparison. Although some authors have proposed intuitively appealing explanations of underlying mechanisms [89,90], residuals in our simulations are determined purely by the dynamic balance of multiple cross-bridge processes (detachment, deformation decay, rebinding, cycling) in a manner that precludes an intuitive generalized explanation. A systematic analysis of these residuals is therefore beyond the scope of the present work.

The energy-cost analysis shown in Fig. 8 summarizes further fundamental distinctions between the LA and LV. Other authors have reported a relatively greater rate of ATP consumption to produce a given amount of force in LA compared to LV [6,15,57]. Our calibrated models support this conclusion qualitatively. In the isometric steady state, the greater force observed in LV is explained primarily by a greater occupancy of the strongly bound state S (Fig. 8(a)). In contrast, LA has a greater detachment rate constant $k_{\text{SU}}$, representing the transition from S to the unbound state U (Fig. 8(b)). Fig. 8(c) shows that the resulting energetic cost (per unit force generated) is 2.3 times greater for LA than LV, in qualitative agreement with the approximate 5-fold difference measured by Narolska et al. [6]. These LA/LV differences can be interpreted within their broader functional context: whereas the LV is required to sustain large forces economically to achieve the required blood ejection against strong afterloads, the crossbridge turnover in LA must be faster to accelerate LV filling, particularly during the “booster” phase of the atrial cycle, albeit at a greater energetic cost. By swapping the model parameters to generate hypothetical hybrid models, Fig. 8(c) further suggests that this LA/LV distinction is explained by the faster transition rate constant $k_{\text{SU}}$ between the strongly bounded and unbound state in LA compared to LV.

At high [ATP], the cross-bridge cycling rate is known to be limited by ADP release, which precedes the more rapid cross-bridge dissociation prompted by ATP binding [91–93]. It is therefore surprising at first sight that our detachment transition S→U should be limiting the cycling rate. However, our schematic model framework omits the multiple stages of the power stroke associated with the release of inorganic phosphate and then ADP [92]. The paradox could therefore be resolved insofar as state S amalgamates the ADP-bound and -unbound states, thereby conferring the rate-limiting function to $k_{\text{SU}}$.

LA/LV differences in cross-bridge properties are likely to arise from underlying regional variations of the two main cardiac myosin isoforms [15,16,94]. Healthy human atria predominantly express α-myosin (~ 75%), whereas ventricles express mostly β-myosin (~ 95%) [15,17,95]. Although both isoforms share the same cross-bridge cycle structure, the measured actin-activated ATPase activity of the α isoform exceeds that of β by a factor of 2 ~ 3 [15]. Given that one ATP molecule is consumed per cross-bridge cycle, the overall cycling rates must therefore differ by the same ratio. We observe a similar difference between our LA and LV $k_{\text{tr}}$ estimates, which correlate with $k_{\text{SU}}$, the rate-limiting step of the cross-bridge cycle in the present model framework. Consistent with the differences noted in Fig. 8, Walklate et al. note that, despite sharing a similar mechanochemical cycle, myosin isoforms differ significantly in terms of the speed of cycling and the fraction of the cycle time spent in the force-generating states [15]. They estimate a 2.86-fold difference in the ATPase rate needed to maintain a 5 pN isometric load on individual myosins (1.0 and 0.35 ATP/s for the α and β isoforms, respectively), consistent with our predicted 2.3-fold difference in force cost (Fig. 8(c)) [15]. Johnson et al. report that ATP binding to the rigor state of human muscle myosin II is almost three times greater for α than β S1 [16], consistent with our determined values for $k_{\text{SU}}$ (Table 2, Fig. 8(b)). Taken together, our results therefore support the differences between human LA and LV dynamics as arising from their specific isoform contents.

The cross-bridge duty ratio, defined as the fraction of actin-bound myosins in steady state, is expected not to exceed ~ 10% in muscle [15]. Within the present model framework, this is represented by the sum $S_{\text{ss}}+W_{\text{ss}}$. This quantity is dictated by the steady-state balance of all the transitions in the model; it is therefore also influenced by the OFF-state force-feedback mechanism, which controls the proportion of myosins available for forming cross bridges. By averaging $S_{\text{ss}}+W_{\text{ss}}$ over 100 MCMC samples (for fixed SL=2.2μm and pCa = 4.5), we obtain 0.053 ± 0.025 for LA and 0.13 ± 0.04 for LV, both consistent with the 10% upper limit but differing by ~ 145%. Since, in the isometric steady state, the active force (Eq. (3)) simplifies to $F_{\text{a},\text{max}}=\frac{T_{\text{ref}}}{r_{\text{S}}}\left(S_{\text{ss}}+W_{\text{ss}}\right)$, this implies that the ~ 50% greater active force predicted in LV compared to LA results from the 145% *increase* in duty ratio $\left(S_{\text{ss}}+W_{\text{ss}}\right)$ dominating the 36% *decrease* in the individual cross-bridge force (quantified by $T_{\text{ref}}/r_{\text{S}}$). This result is consistent with the notion that the average force magnitude observed for different myosin isoforms depends less on the force exerted by single cross bridges than from the fraction of the time spent by the cross bridges in the force-generating states [15].

Ultimately, the importance of these LA/LV differences will become fully appreciated when they are considered in the context of whole-heart function. Faster force-generation dynamics in the LA may favor rapid LV filling and rapid atrial relaxation, allowing efficient and unimpeded LV contraction. Greater force magnitudes and length-dependent response – with greater energetic efficiency – in the LV may be critical for effective ejection into the circulation. Testing these hypotheses will involve the implementation of both LA and LV models jointly within a multi-scale whole-heart simulation. An essential challenge in that endeavor will be the tuning of model parameters to transpose skinned-fiber properties to intact tissue [37]. However, once realized, such whole-organ simulations will constitute a test bench for exploring potential therapeutical applications. These may include mavacamten, an OFF-state stabilizing drug [96–99], and the insulin-based modulation of titin stiffness as a treatment of cardiac myopathies [100–102].

### Limitations

The donor cohort used in this study consisted of 8 males and 1 female (Table S2). Although a properly balanced cohort is always preferable, this requirement is notoriously difficult in practice, with rates of heart donations in the USA being significantly lower for females [103]. The present study focused on samples procured from donors with good organ-level function and providing both atrial and ventricular tissue, thereby limiting choice.

Our analysis sought to identify the models that maximized the likelihood of agreement with the experimental measurements. However, even these “most likely” models display systematic deviations from the measured dynamics (in particular the positive stretch responses in Figs. 4a,b) despite showing reasonable quantitative agreement in terms of the principal components (Figs. 4c,d). Specifically, whereas the experimental dynamic responses feature three decay modes, the simulations reproduce only two. This discrepancy is likely to result from the intrinsic simplicity of our model framework, which assumes a crossbridge cycle with only two actin–myosin bound states. The connection between the structure of the crossbridge cycle scheme and the number of dynamic terms in the response transfer function has been discussed by Kawai [104,105]. A more refined model framework, involving additional crossbridge states would likely yield better overall agreement, albeit at the expense of introducing further unknown parameters.

The calibration procedure probed the force response of cross bridges in the isometric steady state, or near it, in the presence of small length perturbations. Although this approach arguably limited interference from mechanisms associated with more macroscopic perturbations, the applicability of the models to simulations of larger-scale deformations may therefore also be limited, pending a systematic inclusion of appropriate experimental data, e.g., isotonic contraction measurements, in the analysis.

While showing qualitative agreement with the $k_{\text{tr}}$ measurements, both models quantitatively overestimate the experimental values by a factor of ~ 2. Several explanations are possible. One potential source of systematic error is the principal-component-based calibration. Our approach utilizes the principal variations contained within the whole dataset without referring explicitly to specific features. While this method aims to maximize statistical objectivity, the theoretical predictions in Fig. 4 do not accurately resolve the multiphasic response, which introduces some ambiguity in the precise meaning of $k_{\text{tr}}$. Another possibility is the overall simplicity of our model framework, which deliberately involved a minimal number of cross-bridge states. Additional relevant effects are also neglected, including, e.g., cooperativity associated with thin-filament activation as discussed by other authors [106,107]. The possible role of thick-filament OFF/ON dynamics, in itself another kind of cooperativity effect, would merit a more detailed investigation than the present scope allows.

## 5. Conclusion

In summary, the present study is, to our knowledge, the first to present a systematic experimental and biomechanical characterization of the human LA and LV skinned muscle fibers at 37 °C, directly linking sarcomere-level properties to force-generation functionality. The calibrated models demonstrate the ability of thick-filament OFF/ON-state dynamics to account quantitatively for LDA behavior. Factorial analyses performed by hybridizing these two models further demonstrated that the LA/LV differences in LDA (more pronounced in LV) may arise predominantly as consequences of variations in the passive mechanical properties, attributable to the titin molecule. The relatively faster kinetic behavior in the LA arises from the larger values of $k_{\text{SU}}$, which quantifies the flux out of the post-stroke state S. Taken together, these results therefore provide a basis for the further exploration of chamber-specific properties in the context of whole-heart function.

## Supplementary Material

1

## Figures and Tables

**Table 1:** List of acronyms.

LA	left atrium
LV	left ventricle
SL	sarcomere length
LDA	length-dependent activation
MCMC	Markov Chain Monte Carlo
LMER	linear mixed-effects regression
F-pCa	force-calcium relationship
$k_{\text{tr}}$	rate of tension redevelopment
pCa	$-{\text{log}}_{10}\left(\left[{\text{Ca}}^{2+}\right]\right)/\text{mol}\;1^{-1}$

**Table 2:** Model parameters and LA/LV calibration. The full list of model parameters is shown together with the calibrated values for LA and LV, as determined by the MCMC analysis. Parameters marked ~ in the last column were imported directly from the original 2017 Land et al. model [37] without further fitting. Parameters marked * display a significant systematic difference between LA and LV, based on the degree of overlap in the posterior likelihood distributions ℒ[θ] (Fig. 5). The subscripts “ss” denote the isometric steady state.

Model parameters		units	LA		LV	
a	Effective passive stiffness	(kPa)	3.9		6.7	*
$SL_{0}$	Effective resting length	(μm)	1.45		1.61	*
$K_{\text{OFF}}$	Baseline (zero load) OFF/ON state eqb. constant		3.5 × 10^−3^		4.1 × 10^−3^	
$k_{\text{force}}$	OFF/ON state feedback gain	(kPa^−1^)	0.53		0.68	
$T_{\text{ref}}$	Reference force	(kPa)	168		107	
${\text{pCa}}_{\text{T50}}^{\;\text{ref}}$	Reference [Ca^2+^] sensitivity of troponin			6.02		
$n_{\text{TRPN}}$	Troponin Hill coefficient			1.65		
$k_{1}$	OFF → ON transition rate (zero load)	(s^−1^)		2.5×10^−3^		
$n_{\text{Tm}}$	Tropomodulin Hill coefficient			2.0		
$k_{\text{UW}}$	U→W rate constant	(s^−1^)	2.1 × 10^−4^		4.7 × 10^−4^	
$k_{\text{WS}}$	W→S rate constant	(s^−1^)	2.3 × 10^−3^		0.53 × 10^−3^	*
$\gamma_{\text{S}}$	Strain-dependent S→U rate constant	(s^−1^)	409		158	*
$\gamma_{\text{W}}$	Strain-dependent W→U rate constant	(s^−1^)		1.5 × 10^−5^		
ϕ	Distortion decay rate factor		0.93		2.2	*
$A_{\text{eff}}$	Cross-bridge/sarcomere strain ratio		2.9		3.8	*
$r_{\text{W}}$	Partial duty ratio $W_{\text{ss}}/\left(U_{\text{ss}}+W_{\text{ss}}\right)$		0.29		0.39	
$r_{\text{S}}$	Partial duty ratio $S_{\text{ss}}/\left(U_{\text{ss}}+W_{\text{ss}}+S_{\text{ss}}\right)$			0.61		
TRPN50	Value of CaTRPN where B=0.5 in steady state			0.30		
$k_{\text{U}}$	B → U rate constant	(s^−1^)		1000		~
$k_{\text{TRPN}}$	Ca^2+^-troponin binding rate constant	(s^−1^)		100		~
Derived parameters						
$k_{\text{WU}}$	$\text{W}\to \text{U}\;\text{rate}\;\text{constant}=k_{\text{UW}}\times \left(1/r_{\text{W}}-1\right)$	(s^−1^)	4.8 × 10^−4^		7.3 × 10^−4^	
$k_{\text{SU}}$	$\text{S}\to \text{U}\;\text{rate}\;\text{constant}=k_{\text{WS}}\times r_{\text{W}}\left(1/r_{\text{S}}1\right)$	(s^−1^)	441		110	
$k_{\text{B}}$	$\text{U}\to \text{B}\;\text{rate}\;\text{constant}=k_{\text{U}}\times {{\text{TRPN}}_{50}}^{n_{\text{Tm}}}/\left(1-r_{\text{S}}\right)\left(1-r_{\text{W}}\right)$	(s^−1^)	2.1 × 10^−3^		2.5 × 10^−3^	

**Table 3:** Experimental characterizations. (a) The steady-state measurements (F-pCa relationship features in Fig. 2) were computed using the linear mixed-effects regression analysis. (b) The dynamic observables were obtained by projecting the normalized experimental quick-stretch responses $\tilde{F}$ onto the principal component functions (Fig. 3(c), Eq. (2)). The values correspond to the distributions shown in Fig. 3(d). (Projections $c_{8}$ to $c_{12}$ are discarded as they are dominated by measurement noise.) These values, $\mu_{i}$ and $\sigma_{i}$, define the target of the likelihood function (see Supplement). All the evaluations correspond to the average stretch, ${\text{SL}}_{\text{c}}=2.05\;\mu \text{m}$.

Observables		LA	LV	p-values
		$\mu_{i}\pm \sigma_{i}$	$\mu_{i}\pm \sigma_{i}$	
**(a) steady state (F-pCa features)**				
$F_{\text{min}}^{\text{ss}}$	(kPa)	1.68 ± 0.15	1.92 ± 0.19	0.2
$dF_{\text{min}}^{\text{ss}}/d\text{SL}$	(kPa/μm)	2.32 ± 1.05	4.02 ± 0.82	
$F_{\text{a},\text{max}\;}^{\text{ss}}$	(kPa)	11.6 ± 0.9	16.8 ± 1.1	< 0.001
$dF_{\text{a},\text{max}}^{\text{ss}}/d\text{SL}$	(kPa/μm)	7.2 ± 6.3	12.0 ± 4.9	
pCa_50_		5.807 ± 0.011	5.788 ± 0.014	0.2
$d{\text{pCa}}_{50}/d\mu \;\text{m}$	$\left(\mu {\text{m}}^{-1}\right)$	0.061 ± 0.077	0.158 ± 0.060	
$n_{\text{H}}$		2.141 ± 0.065	2.232 ± 0.083	0.3
$dn_{\text{H}}/d\text{SL}$	$\left(\mu {\text{m}}^{-1}\right)$	−0.79 ± 0.47	−0.57 ± 0.36	
**(b) dynamic (principal component projections)**				
$c_{1}$		−4.6 ± 1.6	2.8 ± 3.1	< 0.001
$c_{2}$		−1.1 ± 1.6	−0.5 ± 2.1	0.2
$c_{3}$		−0.7 ± 2.2	0.4 ± 1.7	< 0.001
$c_{4}$		−0.2 ± 1.5	−0.4 ± 1.2	0.1
$c_{5}$		0.08 ± 0.57	−0.26 ± 0.86	0.08
$c_{6}$		−0.14 ± 0.59	0.14 ± 0.55	0.02
$c_{7}$		0.20 ± 0.38	−0.18 ± 0.46	0.001

## Appendix A. Supplementary data

Supplementary material related to this article can be found online at https://doi.org/10.1016/j.yjmcc.2025.12.001.
