# Supplementary material for: Human atrial skinned muscle fibers exhibit reduced length-dependent activation but show faster force development kinetics than ventricular muscle
Source: J Mol Cell Cardiol. Author manuscript; Available in PMC 2026 Jun 26. (PMC13307878; doi:10.1016/j.yjmcc.2025.12.001)
Supplement: 1 [file NIHMS2187426-supplement-1.pdf]

Human atrial skinned muscle fibers exhibit reduced length-dependent activation but show faster force development kinetics than ventricular muscle SUPPLEMENT

Alexandre Lewalle, Gregory Milburn, Jania Bell,

Kenneth S Campbell, Steven A Niederer

S1 Experimental details

S1.1 Tissue samples

A total of 126 muscle samples were prepared from the LA and LV of a cohort of 9 donors not suffering from cardiovascular disease. Each sample prep was stretched to a sarcomere length of either 1.94 ± 0.03 μm or 2.23 ± 0.03 μm. Table S1 shows the distribution of the samples for each region and each sarcomere length.

Measurements were performed for each preparation at the pCa values of 9.0, 6.6, 6.4, 6.2, 6.0, 5.8, 5.6, 5.4, 5.0, and 4.5.

Table S1. Number of measured procured samples

	Patient	LA		LV	
		1.9 μm	2.2 μm	1.9 μm	2.2 μm
1	2508D	3	4	4	6
2	2B487	6	3	4	6
3	2DDC0	4	3	3	0
4	30B2B	2	10	3	6
5	5155D	1	0	5	1
6	5245C	2	2	3	3
7	B23E3	4	3	5	4
8	BC90C	3	3	4	4
9	BE318	4	2	3	3
	totals	29	30	34	33

Table S2. Patient demographics

Patient ID	Sex	Cause of Death	Age (yrs)	BMI (kg/m^2)	HbA1c (%)	LVEF (%)	LVPWd (cm)	LVIDd (cm)	ECG rhythm
2508D	Male	CVA/Stroke	40.7	32.1	5.3	31	1.8	4.6	Sinus
2B487	Male	Traumatic brain injury	41.6	31	5.3	55	1.0	4.1	Sinus
2DDC0	Male	Traumatic brain injury	48.5	24.4	5.2	56	0.8	4.6	Sinus
30B2B	Male	Anoxic brain injury; unknown etiology	71.2	24.2	5.2	66	1.2	4.0	-
5155D	Male	Anoxic brain injury; unknown etiology	35.7	22.2	9.9	53	1.1	4.7	Sinus
5245C	Male	CVA/Stroke	55.3	19.7	4.8	-	-	-	-
B23E3	Male	CVA/Stroke	50.1	29.4	5.5	55	1.0	4.2	Sinus
BC90C	Female	Anoxic brain injury; drug overdose	38.6	24.1	5.2	55	1.0	4.4	Sinus
BE318	Male	CVA/Stroke	45.7	24.5	7.0	70	1.0	4.8	Sinus
Mean ± SD			47.5 ± 10.8	25.7 ± 4.2	5.9 ± 1.6	55.1 ± 11.5	1.1 ± 0.3	4.4 ± 0.3	

S1.2 Experiment solutions

Three different batches of solutions were prepared with the same recipes and reagents. We sampled from the different experimental groups randomly such that no solution batch was used exclusively for a single experimental group.

The compositions of the solutions used in the experiments were as follows:

Relaxing solution

KCl 100 mM, Imidazole 20mM, EGTA 2mM, ATP 4 mM, $MgCl_2$ 7 mM.

All chemicals were purchased from Sigma-Aldrich, except stock $MgCl_2$.

pCa solutions

The precise composition of each pCa solution was determined using Maxchelator software [1]. pCa solutions for intermediate calcium concentrations were made by diluting the high pCa solution with low pCa solution, prepared as follows:

pCa 4.5: EGTA 7 mM (Sigma), BES 50 mM (Sigma), K propionate 32 mM (TCI), creatine phosphate 14.5 mM (Roche), ATP 4.64 mM (Roche), DTT 1 mM (Roche), $CaCl_2$ 7.01 mM (Orion), $MgCl_2$ 5.21 mM (Stock). pH 7.

pCa 9: EGTA 7 mM (Sigma), BES 50 mM (Sigma), K propionate 48 mM (TCI), creatine phosphate 14.5 mM (Roche), ATP 4.58 mM (Roche), DTT 1 mM (Roche), $CaCl_2$ 0.0186 mM (Orion), $MgCl_2$ 5.68 mM (Stock). pH 7.

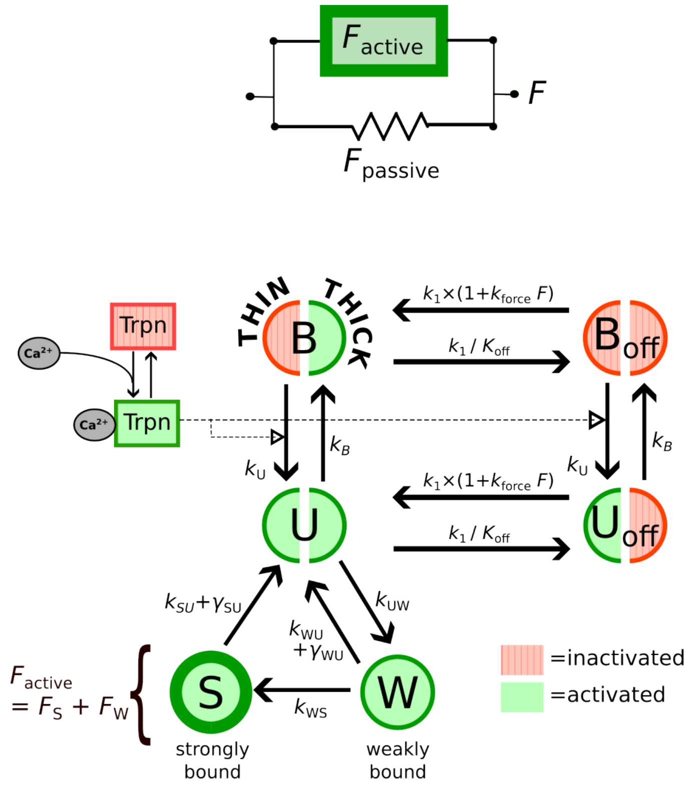

Figure S1. Theoretical model framework. (a) Overall model of the force-generation scheme, consisting of a passive elastic element placed in parallel with the active contraction force. (b) The kinetic scheme for active force generation. Each state symbol represents the activation status of the thin (left shell) and thick filaments (right shell). In the B and U states, the thin filaments are, respectively, inactivated and activated by calcium-troponin, with the thick filaments in the "ON" state. B_{off} and U_{off} correspond to the inactivated thick filaments, the "OFF" state. Filament binding leads from U to the weakly bound state W and the myosin power stroke transitions to the strongly bound state S. The transition rate constants define the ODE system.

S2 ODE system

S2.1 Equations

The present model inherits the ODEs of the original Land model [2], amended to include additional terms representing the transitions into and out of the new OFF states B_{off} and

$U_{\rm off}$ in Fig. S1:

$$\begin{aligned}
\frac{dB}{dt} &= k_{\rm B}\cdot {\rm CaTRPN}^{-n_{\rm Tm}/2}\cdot U - k_{\rm U}\cdot {\rm CaTRPN}^{n_{\rm Tm}/2}\cdot B \\
&\quad + k_1(F_{\rm total})\cdot B_{\rm off} - k_2\cdot B \qquad \text{(S1)}\\
\frac{dB_{\rm off}}{dt} &= k_{\rm B}\cdot {\rm CaTRPN}^{-n_{\rm Tm}/2}\cdot U_{\rm off} - k_{\rm U}\cdot {\rm CaTRPN}^{+n_{\rm Tm}/2}\cdot B_{\rm off} \\
&\quad + k_2\cdot B - k_1(F_{\rm total})\cdot B_{\rm off} \qquad \text{(S2)}\\
\frac{dU_{\rm off}}{dt} &= -k_{\rm B}\cdot {\rm CaTRPN}^{-n_{\rm Tm}/2}\cdot U_{\rm off} + k_{\rm U}\cdot {\rm CaTRPN}^{+n_{\rm Tm}/2}\cdot B_{\rm off} \\
&\quad + k_2\cdot U - k_1\cdot U_{\rm off} \\
\frac{dW}{dt} &= k_{\rm UW}U - k_{\rm WU}W - k_{\rm WS}W - \gamma_{\rm WU}(\zeta_{\rm W})W \\
\frac{dS}{dt} &= k_{\rm WS}W - k_{\rm SU}S - \gamma_{\rm SU}(\zeta_{\rm S})S \\
U &= 1 - B - W - S - B_{\rm off} - U_{\rm off} \qquad \text{(S3)}
\end{aligned}$$

where CaTRPN is the proportion of calcium-bound troponin-C units

$$d{\rm CaTRPN}/dt = k_{\rm TRPN}\left[\left([{\rm Ca}^{2+}]/[{\rm Ca}^{2+}]_{50}^{\rm Trpn,ref}\right)^{n_{\rm TRPN}}(1-{\rm CaTRPN}) - {\rm CaTRPN}\right],$$

and $k_{\rm U}$, $k_{\rm UW}$, $k_{\rm WU}$, $k_{\rm WS}$, $k_{\rm SU}$, and $k_{\rm TRPN}$ are transition rate constants. The OFF-state feedback mechanism is implemented through a force dependence of the OFF-ON transition rate [3]:

$$k_1(F_{\rm total}) = k_1^0(1 + k_{\rm force}F_{\rm total}) \qquad \text{(S4)}$$

Following the original Land model, the remaining rate constants are derived parameters calculated using these basic rate constants and the duty ratios as

$$\begin{aligned}
k_{\rm B} &= k_{\rm U}\ {\rm TRPN_{50}}^{n_{\rm Tm}}/[(1-r_{\rm S})(1-r_{\rm W})] \\
k_{\rm WU} &= k_{\rm UW}\ (1/r_{\rm W} - 1) - k_{\rm WS} \qquad \text{(S5)}\\
k_{\rm SU} &= k_{\rm WS}\ r_{\rm W}(1/r_{\rm S} - 1) \qquad \text{(S6)}
\end{aligned}$$

with

$$\begin{aligned}
r_{\rm W} &= W_{\rm ss}/(W_{\rm ss} + U_{\rm ss}) \\
r_{\rm S} &= S_{\rm ss}/(S_{\rm ss} + W_{\rm ss} + U_{\rm ss})
\end{aligned} \qquad \text{(S7)}$$

representing “partial duty ratios” that relate the corresponding steady state (‘ss”) popu-

lations. It follows from Equation S7 that, in the steady state,

$$W_{ss} = U_{ss} \times r_W/(1 - r_W) \tag{S8}$$

$$S_{ss} = U_{ss} \times r_S/(1 - r_S)(1 - r_W) \tag{S9}$$

The terms $\gamma_{WU}(\zeta_W)$ and $\gamma_{SU}(\zeta_S)$ represent cross-bridge unbinding rate constants for the W and S states, and are assumed to depend on the cross-bridge distortions ζ_W and ζ_S. These distortions are treated as state variables (in addition to the state populations), obeying

$$\begin{aligned} d\zeta_W/dt &= A\ d\lambda/dt - c_W\ \zeta_W \\ d\zeta_S/dt &= A\ d\lambda/dt - c_S\ \zeta_S \end{aligned} \tag{S10}$$

where

$$\lambda = \mathrm{SL}/\mathrm{SL}_0 \tag{S11}$$

is the relative cell length relative to the resting length, A is a constant relating the change in sarcomere length to cross-bridge distortion, and the distortion decay rate constants c_W and c_S are assumed to be proportional to the steady-state cross-bridge cycling rates under isometric conditions:

$$\begin{aligned} c_W &= \phi\ k_{UW} \cdot U_{ss}/W_{ss} \\ c_S &= \phi\ k_{WS} \cdot W_{ss}/S_{ss} \end{aligned}$$

where ϕ is a constant. The cross-bridge detachment rate constants (in dW/dt and dS/dt above) are then given by

$$\begin{aligned} \gamma_{WU}(\zeta_W) &= \gamma_W\ |\zeta_W| \\ \gamma_{SU}(\zeta_S) &= \begin{cases} -\gamma_S(1+\zeta_S) & \zeta_S < -1 \\ 0 & \zeta_S \in [-1, 0] \\ \gamma_S \zeta_S & \zeta_S > 0 \end{cases} \end{aligned} \tag{S12}$$

The total average force exerted by the cross bridges is given by the combination of the passive force (assumed here to be linear) and active force (following the original Land

model [2]):

$$F_{\text{passive}} = a\left(\frac{\text{SL}}{\text{SL}_0} - 1\right) \quad \text{(S13)}$$

$$F_{\text{active}} = \frac{T_{\text{ref}}}{r_{\text{S}}}[(\zeta_{\text{S}}+1)S + \zeta_{\text{W}}W] \quad \text{(S14)}$$

$$F_{\text{total}} = F_{\text{active}} + F_{\text{passive}} \quad \text{(S15)}$$

S2.2 Closed-form solution of the ODE system in the isometric steady state

The isometric steady state ("ss") of the model is determined by equating the left hand side of each ODE to zero for constant λ. The relative populations of the states are given by the equilibrium constants:

$$\begin{aligned} K_{\text{UB}} &= \frac{U_{\text{ss}}}{B_{\text{ss}}} = \frac{U_{\text{off,ss}}}{B_{\text{off,ss}}} = \frac{k_{\text{U}}}{k_{\text{B}}}\ \text{CaTRPN}_{\text{ss}}^{+n_{\text{Tm}}} \\ K_{\text{OFF}} &= \frac{U_{\text{ss}}}{U_{\text{off,ss}}} = \frac{B_{\text{ss}}}{B_{\text{off,ss}}} \\ &= \frac{k_1(F_{\text{total}})}{k_2} = K^0_{\text{OFF}} \times [1 + k_{\text{force}}\,(F_{\text{active,ss}} + F_{\text{passive,ss}})] \end{aligned} \quad \text{(S16)}$$

Hence, we have

$$U^{\text{ss}}_{\text{off}} = \frac{1}{K_{\text{OFF}}[1 + k_{\text{force}}(1 + k_{\text{force}}F_{\text{total,ss}})]}\ U_{\text{ss}} \quad \text{(S17)}$$

$$B^{\text{ss}}_{\text{off}} = \frac{1}{K_{\text{UB}}}\ U^{\text{ss}}_{\text{off}} \quad \text{(S18)}$$

$$B_{\text{ss}} = \frac{1}{K_{\text{UB}}}\ U_{\text{ss}} \quad \text{(S19)}$$

$$W_{\text{ss}} = \frac{k_{\text{UW}}}{k_{\text{WU}} + k_{\text{WS}}}\ U_{\text{ss}} \quad \text{(S20)}$$

$$S_{\text{ss}} = \frac{k_{\text{WS}}}{k_{\text{SU}}} \cdot \frac{k_{\text{UW}}}{k_{\text{WU}} + k_{\text{WS}}}\ U_{\text{ss}} \quad \text{(S21)}$$

$$\zeta^{\text{ss}}_{\text{S}} = 0 \quad \text{(S22)}$$

$$\zeta^{\text{ss}}_{\text{W}} = 0 \quad \text{(S23)}$$

$$\text{CaTRPN}_{\text{ss}} = \frac{([\text{Ca}^{2+}])^{n_{\text{TRPN}}}}{([\text{Ca}^{2+}])^{n_{\text{TRPN}}} + ([\text{Ca}^{2+}]_{50}^{\text{Trpn,ref}})^{n_{\text{TRPN}}}} \quad \text{(S24)}$$

In the isometric steady state, the active force (Equation S14) reduces to

$$\begin{aligned} F_{\text{active,ss}} &= \frac{T_{\text{ref}}}{r_{\text{S}}} S_{\text{ss}} \\ &= \underbrace{\frac{T_{\text{ref}}}{r_{\text{S}}} \cdot \frac{k_{\text{WS}} k_{\text{UW}}}{k_{\text{SU}}(k_{\text{WU}} + k_{\text{WS}})}}_{\mu} U_{\text{ss}} \end{aligned} \tag{S25}$$

Applying the conservation constraint $B_{\text{ss}} + B_{\text{off,ss}} + U_{\text{ss}} + U_{\text{off,ss}} + W_{\text{ss}} + S_{\text{ss}} = 1$ and substituting the equilibrium conditions derived above, we obtain

$$U_{\text{ss}} = \left[\left(1 + \frac{1}{K_{ub}}\right) \left(1 + \frac{1}{K_{\text{OFF}}}\right) + Q \right]^{-1} \tag{S26}$$

where

$$\begin{aligned} Q &= \frac{k_{\text{WS}} k_{\text{UW}}}{k_{\text{SU}}(k_{\text{WU}} + k_{\text{WS}})} + \frac{k_{\text{UW}}}{k_{\text{WU}} + k_{\text{WS}}} \\ &= \frac{r_{\text{S}} + r_{\text{W}} - r_{\text{S}} r_{\text{W}}}{(1 - r_{\text{S}})(1 - r_{\text{W}})} \end{aligned}$$

The force dependence of U_{ss} is contained implicitly in K_{OFF} via Equation S16. Substituting Eqs. S16 and S25 into Eq. S26 yields a quadratic equation for U_{ss}, with solution

$$U_{\text{ss}} = \frac{\beta \left[-1 + \left(1 + \frac{4\mu \left(1 + \frac{1}{K_{\text{UB}}} + Q\right)\left(1 + k_{\text{force}} F_{\text{passive}}\right)}{\beta^2}\right)^{1/2} \right]}{2\mu \left(1 + \frac{1}{K_{\text{UB}}} + Q\right)} \tag{S27}$$

where

$$\beta = (1 + k_{\text{force}} F_{\text{passive}}) \left(1 + \frac{1}{K_{\text{UB}}} + Q\right) + \frac{1}{K^0_{\text{OFF}}} \left(1 + \frac{1}{K_{\text{UB}}}\right) - \mu$$

and μ is the constant prefactor defined in Eq. S25.

Under isometric conditions, the steady-state solution for the total force,

$$F_{\text{total,ss}} = F_{\text{active,ss}} + F_{\text{passive,ss}} \tag{S28}$$

is hence obtained by substituting Eq. S27 into Eq. S25. This solution is implicitly [Ca^{2+}]-dependent via the state variable CaTRPN (see [2] and SL-dependent via $F_{\text{passive,ss}}$).

Model code

Computer code for the calibrated model implementations can be found at
`https://github.com/AlexLewalle/LA_LV_models` .

S3 Parameter calibration procedure

S3.1 Likelihood function

For a given test model represented by a parameter set θ, a likelihood function is computed from the observables' mean values μ_i and uncertainties σ_i:

$$\mathcal{L}[\theta] = \prod_i \frac{\sqrt{w_i}}{\sigma_i\sqrt{2\pi}} \exp\left[-\frac{(m_i[\theta]-\mu_i)^2}{2\sigma_i^2}\cdot w_i\right] \times \mathcal{L}_{\text{trace}}[\theta] \tag{S29}$$

where $m_i[\theta]$ denotes the test-model prediction of the corresponding μ_i, w_i are weighting factors, and $\mathcal{L}_{\text{trace}}[\theta] = \langle(\tilde{F}(t) - \tilde{F}_{\text{target}}(t))^2/2\sigma_{\text{target}}(t)^2\rangle$ represents an additional cost term that further minimizes the residue between the predicted dynamic force trace $\tilde{F}$ and the target (experimental) traces $\tilde{F}_{\text{trace}}$. Although the optimization of $\mathcal{L}[\theta]$ by MCMC (achieved computationally by maximizing $\log(\mathcal{L}[\theta])$; see below) formally resembles a least-squares minimization, this approach additionally provides a statistical handle for comparing the significance of LA/LV parameter differences in terms of their posterior distributions.

The steady-state observables used for μ_i were $F_{\text{a,max}}$, F_{min}, pCa_{50}, and n_{H}, $dF_{\text{a,max}}/d\text{SL}$, $dF_{\text{min}}/d\text{SL}$, and $d\text{pCa}_{50}/d\text{SL}$ returned by the LMER analysis. We did not include $dn_{\text{H}}/d\text{SL}$ because of its low statistical significance (high p value, see Figure 2(b) in the main paper), in order to allow a better resolution of the other gradients. For the dynamic observables, we used the coefficients c_j ($1 \leq j \leq 7$) not dominated by experimental noise, taken from the projections of the kinetic traces onto the principal components for quick stretches of $\pm 1\%$ done at SL = 1.9 μm.

We set $w_i = 1$ for all the observables except for the c_j terms, which were weighted according to the "proportion of explained variance", as determined by the principal component analysis (see Figure 3(b) in the main paper). This weighting scheme was designed to avoid biasing the fitting outcomes by c_j components representing negligible variations in the data. The μ_i and σ_i values used for calibration, as obtained from the experimental steady-state and dynamic data analyses, are listed in Table 3 of the main manuscript.

S3.2 Preliminary parameter estimates

Preliminary parameter values were estimated as follows. Following an approach described previously [3], we used a Nelder-Meade optimization to determine the values of a, SL_0, K_{OFF}, k_{force}, T_{ref}, $\mathrm{pCa}^{\mathrm{ref}}_{\mathrm{T50}}$, and n_{TRPN} that matched the steady-state LDA observables, while initially assuming the Land model values for r_{W}, r_{S}, TRPN_{50}, and n_{Tm} [2]. We then generated a cohort of 10^6 test models sampled from a Latin hypercube spanning three orders of magnitude of k_1, k_{U},k_{UW}, k_{WS}, γ_{W}, γ_{S}, ϕ, and A_{eff}, taking the Land model values as a central estimate. For each test model, we simulated $\tilde{F}$, extracted the c_j coefficients (Equation S30), and computed the corresponding likelihoods using Equation S29. The parameter set yielding the maximum likelihood was then used to define the initial values in the subsequent MCMC analysis.

S3.3 Markov-Chain Monte Carlo calibration

Markov Chain Monte Carlo analysis was used to estimate the posterior parameter distributions that map onto the observables' distribution [4–6]. This involved defining a set of 36 "walkers" (2× the number of investigated parameters), initially randomly distributed within 10% of the preliminary fit values. The walkers then propagated within the parameter space θ, guided by the likelihood function $\mathcal{L}[\theta]$ (Equation S29). The upper bound on individual parameters was typically set to 10 times the preliminary estimate. (See Supplement for implementation details.)

The MCMC algorithm outputs the trajectories of the walkers in θ space together with the associated likelihood values $\mathcal{L}[\theta]$. We estimated the parameter posterior distributions by binning each parameter range and identifying the parameter values (θ coordinates) with the maximum likelihood contained in each bin. By this method, the true posterior distributions gradually emerge as the MCMC walker trajectories approach the steady state.

The MCMC algorithm outputs the trajectories of the walkers in θ space together with the associated likelihood values $\mathcal{L}[\theta]$. We estimated the parameter posterior distributions by binning each parameter range and identifying the parameter values (θ coordinates) with the maximum likelihood contained in each bin. By this method, the true posterior distributions gradually emerge as the MCMC walker trajectories approach the steady state.

S4 Analysis tools

S4.1 Linear mixed-effects regression

The analysis assumes each measurement to be the sum of a fixed effect, a random sample-wise variation, and an additional random patient-wise variation. The algorithm accommodates inhomogeneities and gaps in the dataset (an often inevitable feature of the experimental measurements) and optimally determines the underlying effect and its associated uncertainty. This output represents the best estimate of the length dependence shared by all the patients in the cohort.

The analysis was performed using the python `statsmodel` package. For the purpose of the model calibrations, we aimed to fit the value of each observable($y = F_{\mathrm{a,max}}$, F_{min}, pCa_{50}, or n_{H}) and the associated derivative ($dy/d\mathrm{SL}$), evaluated at a central sarcomere length $\mathrm{SL_c} = \langle \mathrm{SL} \rangle$. We treated the sarcomere length as a continuous variable and the cardiac region (LA or LV) as a categrical variable, grouped patient-wise.

The fitting algorith was invoked by using the `statsmodels.formula.api.mixedlm` function, specifying the fixed effects with the `Patsy` formula [7]

```
y ~ 0 + yname + yname:Region + yname:Region:I(SL-meanSL)
```

where `yname` denotes the observable name, `Region` specifies LA or LV, and `meanSL` is the mean sarcomere length. The regression outcomes returned by the algorithm are the values y and $dy/d\mathrm{SL}$, evaluated at the mean SL, with the associated uncertainties and p values.

Code

The corresponding code section for the fitting is summarized as follows:

```
import statsmodels.api as sm
import statsmodels.formula.api as smf
from statsmodels.tools.sm_exceptions import ConvergenceWarning
meanSL = np.mean(PhenDF['SL'])
SL_ = f'I(SL-{meanSL:.2f})'
lmm_fit = smf.mixedlm(
    formula = f"y ~ 0 + yname + yname:Region + yname:Region:{SL_} ",
```

```
        data = PhenDF_global.astype(float).reset_index(),
        groups=PhenDF_global.reset_index()['Patient']).fit(
        method='bfgs')
print(lmm_fit.summary())
```

An alternative `Patsy` formulation of the regression formula allows a more direct evaluation of the mean values of the observables at each specific sarcomere length:

`y ~ 0 +  Region + C(ifloSL, Treatment(reference={ifloSL_ref})):Region`

where the parameter `ifloSL` specifies which of the two sarcomere lengths (1.9 or 2.2 μm), now considered as a categorical variable, to treat as reference.

The following table summarizes the mean values of the observables at each of the two sarcomere lengths, their corresponding uncertainty, and the associated p-values.

	LA			LV		
	1.9 μm	2.2 μm	p	1.9 μm	2.2 μm	p
$F_{\mathrm{a,max}}$	10.52 ± 1.17	12.60 ± 1.34	0.197	14.95 ± 1.04	18.86 ± 1.07	0.003
F_{min}	1.40 ± 0.19	2.07 ± 0.21	0.015	1.35 ± 0.16	2.58 ± 0.17	0.000
n_{H}	2.25 ± 0.10	2.00 ± 0.11	0.064	2.32 ± 0.09	2.17 ± 0.09	0.182
pCa50	5.80 ± 0.02	5.82 ± 0.02	0.276	5.77 ± 0.01	5.81 ± 0.01	0.013

We emphasize that the data points, taken individually cannot be considered as independent measurements because of the uneven distribution of the measurements with respect to the human donors. The purpose of the LMER analysis was therefore to resolve the systematic underlying trends in the data while taking into account the inherent patient-based hierarchical structure of the datasets.

S4.2 Principal component analysis

We performed a principal component analysis (PCA) via a singular value decomposition of the ensemble of all the $\tilde{F}(t)$ traces using the `PCA` function of the `python sklearn.decomposition` package to identify the $n = 12$ most significant principal components $\psi_j(t)$ ($j \in [0, n]$) in the overall dataset [8]. In effect, this procedure reduces the 1000-fold dimensionality of the $\tilde{F}$ vector space (1000 time points per trace) to 12. Each data trace can hence be

formally represented as a low-dimensional vector $\{c_j\}$ via the decomposition

$$\tilde{F}(t) = \langle \tilde{F}(t) \rangle + \sum_{j=1}^{n} c_j \times \ \psi_j(t) + \text{residues} \tag{S30}$$

where $\langle \tilde{F}(t) \rangle$ denotes the mean of all the $\tilde{F}(t)$ functions and the "residues" the total remaining higher-order contributions to $\tilde{F}(t)$, assumed to be negligible. This then provides a handle for comparing the LA and LV dynamics in terms of their respectiv c_j values.

Code

```
def MakeQSBases(n_pts=1001, tmax = 0.45, n_components=5, maxpCa=7.9,
        maxabsdfracdSL=0.02, ifEnd45=False, ifPlot=False):
    from scipy.interpolate import interp1d
    t = np.linspace(0., tmax, n_pts)
    Y = {+1: [], -1: []}
    QSTrace_all = []
    for jRun, Run1 in enumerate(MakeDataIndex()):
        if ifContainsSLCData(Run1) == False:
            print(f'{Run1} contains no data => skipping')
            continue
        print(f'Processing for MakeQSBases ({jRun}): {Run1}')
        DR1 = DataRun_class(Run1)
        QSTrace1 = DR1.Make_QSP_traces(n_pts=n_pts, tmax = tmax,
             maxpCa=maxpCa,  maxabsdfracdSL=maxabsdfracdSL,
             ifEnd45=ifEnd45, ifPlot=False)
        QSTrace_all += list(QSTrace1.to_numpy())
    QSTrace_all = [temp for temp in QSTrace_all
         if not np.isnan(temp).any()]

    QSBasesPCA = PCA(n_components=n_components)
    QSBasesPCA.fit( QSTrace_all) #[np.concatenate( [Y[+1][j] , Y[-1][j]] )
         for j in range(len(Y[+1]))] )
    QSBasesPCA.maxpCa = maxpCa
```

```
QSBasesPCA.tmax = tmax
QSBasesPCA.n_pts = n_pts
QSBasesPCA.n_components = n_components
return QSBasesPCA
```

S4.3 Markov-Chain Monte Carlo (MCMC)

We used the MCMC implementation provided by the python `emcee` package [6]. We created 34 "walkers" (2× the number of investigated parameters) as the initial parameter sets, located in the vicinity of the preliminary estimates. Starting from these initial values, the walkers were made to progress over 10^5 steps according to the Metropolis-Hastings algorithm, implemented in `emcee.EnsembleSampler`. We assumed a flat prior distribution for the parameters, bounded between 0.01 and 10 times the initial values. When the walkers' trajectories eventually reach a steady state, they effectively sample the corresponding posterior distributions, constrained by the experimental data and uncertainties. The algorithm output consists of the sequence of the parameter values over steps s for each walker w, denoted $\theta_w(s)$, with the corresponding likelihood values $\mathcal{L}[\theta_w(s)]$.

The MCMC-generated results were analysed to determine the likelihood profile for each model parameter. For a number of bins covering each parameter range, we used the python function `scipy-stats.binned_statistic` to identify the parameter set $\theta_{\max}$ that, among the combined outputs from all the MCMC walkers, had the maximum likelihood $\mathcal{L}[\theta_{\max}]$. The resulting histograms thus help to identify not only the model (θ_{best}) with the maximum overall likelihood, but also the spread of parameter values whose likelihoods lie within the corresponding likelihood range around $\mathcal{L}[\theta_{\text{best}}]$. The analysis was repeated to produce, in turn, the histograms for LA and LV.

S5 k_{tr} sensitivity analysis

The relative sensitivity of k_{tr} to each basic model parameter p was defined as

$$S_{k_{\text{tr}}}(p) = \frac{\partial k_{\text{tr}}}{\partial p} \times \frac{p}{k_{\text{tr}}} \tag{S31}$$

This was calculated locally by applying a 1% change to each p relative to its calibrated value.

We note that, following the formalism of the original Land model [2], the basic parameter k_{WS} determines the rate constant of two different transitions: W→S (wih rate constant equal to k_{WS}) and S→U (via the derived rate constant $k_{\mathrm{SU}} = k_{\mathrm{WS}} \times r_{\mathrm{W}}(1/r_{\mathrm{S}} - 1)$ – Equation S6). Similarly, k_{UW} determines both U→W and W→U via $k_{\mathrm{WU}} = k_{\mathrm{UW}}\ (1/r_{\mathrm{W}} - 1) - k_{\mathrm{WS}}$ (Equation S5). Therefore, to calculate the effective $S_{k_{\mathrm{tr}}}$ with respect to these transitions specifically, it is necessary to vary each transition rate in isolation, ensuring the others remain fixed.

We obtain $S_{k_{\mathrm{tr}}}[W \to S]$ through an adaptation of Equation S31 by varying k_{WS} (a "derived parameter") while adjusting r_{S} to keep k_{SU} intact:

$$\begin{aligned} k_{\mathrm{WS}} &\to k_{\mathrm{WS}} + \delta k_{\mathrm{WS}} \\ r_{\mathrm{S}} &\to r_{\mathrm{S}} + r_{\mathrm{S}}(1 - r_{\mathrm{S}})/k_{\mathrm{WS}} \times dk_{\mathrm{WS}} \end{aligned}$$

Equation S31 is therefore implemented for this specific transition by applying both of these transformations as part of "∂p".

Likewise, to obtain $S_{k_{\mathrm{tr}}}[S \to U]$ by varying k_{SU} independently of k_{WS}, we differentiate Equation S6 analytically and apply:

$$\begin{aligned} k_{\mathrm{WS}} &\to k_{\mathrm{WS}} \\ r_{\mathrm{S}} &\to r_{\mathrm{S}} - r_{\mathrm{S}}^2/k_{\mathrm{WS}} r_{\mathrm{W}} \times dk_{\mathrm{SU}} \end{aligned}$$

S6 Variance Sensitivity analysis

A sensitivity analysis was performed for the experimental observables with respect to each of the model parameters. We used the variance-based Saltelli metric S_{T} that quantifies the total effect of each parameter on the observable [9]. The implementation, summarized in the Code section below, exploits Gaussian Process Emulators as surrogates for the observables to accelerate the computational task [10].

The computed sensitivity results are shown as a color map in Fig. S2(a). To better summarize the results, we identified, for each parameter, the maximum value of S_{T} across

all the observables. This allows a ranking of the parameters according to their relative significance in determining the observables overall.

Code

```
def DoGSA(Cohort, Emulators, WhichFeatures=[], n_draws=100*len(IncludeParams),
     ifPlot=False, ifFocusParam=False):
    #%%%% Initialise
    GSA_DF = pd.DataFrame(index=WhichFeatures, columns=Cohort.IncludeParams)
    CumulST = np.array([0.]*len(Cohort.IncludeParams))

    #%%%% Compute GSA
    tic = time.time()
    X_train = Cohort.PSetDF[Cohort.IncludeParams].to_numpy()
    for iFeat, Feat1 in enumerate(WhichFeatures):
        print(f'Starting GSA for {Feat1} ')
        dataset = Dataset(X_train=X_train,
                y_train=Cohort.SimResults[Feat1],
                l_bounds=[Cohort.ParRange[param1][0]
                                    for param1 in Cohort.IncludeParams],
                u_bounds=[Cohort.ParRange[param1][1]
                     for param1 in Cohort.IncludeParams])
        gsa1 = SobolGSA(dataset, n=128, seed=GPEseed)
        gsa1.estimate_Sobol_indices_with_emulator(
            Emulators[Feat1], n_draws=n_draws)
        print(f'   GSA complete for {Feat1}')
        gsa1.summary()
        GSA_DF.iloc[iFeat] = np.mean(gsa1.ST, axis=0)

    if ifPlot:
        ShowGSA(GSA_DF)
    return GSA_DF
```

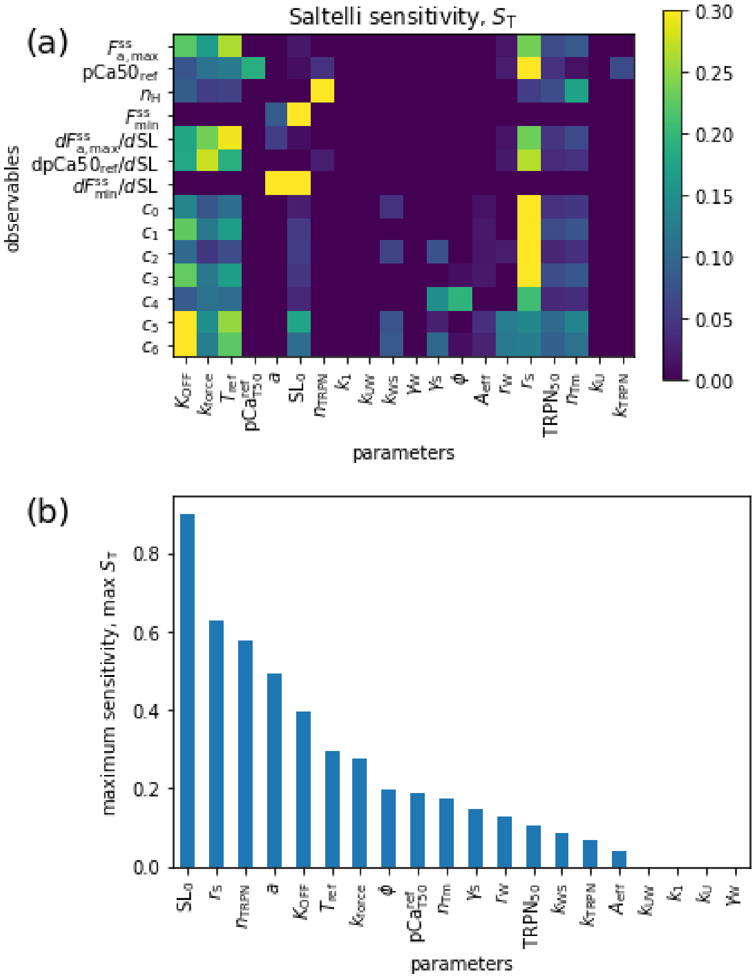

Figure S2. Sensitivity analysis of experimental observables. (a) Color map of the Saltelli sensitivity S_T evaluated for each model parameter [9]. (b) Ranking of the model parameters in terms of the maximum value of S_T.

S7 Supplementary simulations

S7.1 Assessing the impact of neglecting nonlinearity in $F_{\text{passive}}(\text{SL})$

Nonlinearity in the passive mechanical force $F_{\text{passive}}(\text{SL})$ is well reported and often expressed by an exponential function [2, 11, 12]. Resolving this nonlinearity in the present

study would have been feasible in principle by doing measurements at additional sarcomere lengths. We considered the hypothesis that this more thorough characterization of the passive mechanics would not impact our overall results significantly, as follows.

On the one hand, two fixed sarcomere lengths suffice to calibrate the steady-state parameters unambiguously, and therefore all the model predictions relating to the isometric steady state at the two chosen sarcomere lengths (1.9 and 2.2 μm) must remain exact regardless of any nonlinearity.

On the other hand, nonlinearity in $F_{\text{passive}}(\text{SL})$ may in principle affect the force response involving small length changes in the near vicinity (within 1%) of the two set lengths. However, we expect these effects to be higher-order. To test this, we introduced a hypothetical nonlinear dependence into the passive models using the form assumed by Land et al. [2],

$$F_{\text{passive}}^{\text{NONLINEAR}}(\text{SL}) = \alpha \left(e^{\beta(\text{SL}/\text{SL}_0 - 1)} - 1 \right), \tag{S32}$$

constraining its values at 1.9 and 2.2 μm to match those of the linear model (see Figure (a) below). This ensures that the steady-state predictions at these lengths are identical for both models. The extent of nonlinearity was set so as to decrease the estimated slack length by 20%, reaching beyond the expected physiological limit.

The impact on the corresponding dynamic response (Figure S3(b)) is relatively small when considered in terms of the principal component projections used for the calibration process (Figure S3(c)). We conclude that the assumption of linear passive mechanics is expected to have a minor effect on our model calibrations and should therefore not affect our overall analysis significantly.

S7.2 Comparing contributions of active and passive force to cross-bridge recruitment

Our model framework assumes OFF/ON dynamics as the basis of LDA, realized via the force feedback acting on the OFF/ON state balance. In our previous paper [3], we showed that, under this assumption, passive force provides the necessary initial length dependence that then becomes amplified by the feedback. However, this criterion does not imply that the passive mechanics fully determines the LDA behavior quantitatively. Indeed, as we previously showed [3], the feedback input is the total tension and not just the passive force. The precise role of passive mechanics in differentiating between tissue types is

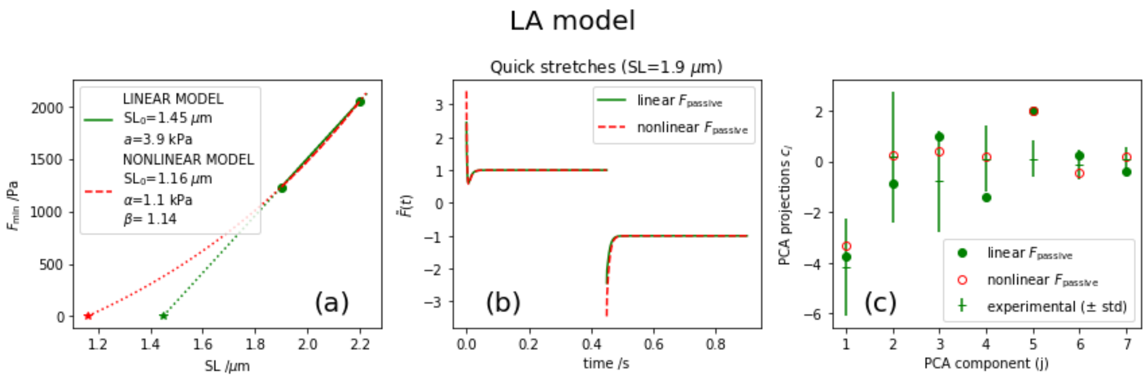

Figure S3. Testing the impact of the linear passive stiffness assumption. (a) A hypothetical model was generated assuming nonlinear $F_{\text{passive}}^{\text{NONLINEAR}}(\text{SL})$ (see Equation S32) with its values at SL $=$ 1.9 and 2.2 μm anchored to the linear-model predictions. (b) Quick-stretch responses, as used in our model calibration procedure, were simulated for both the linear and nonlinear models. (c) Comparison of the principal component projections (as used to define the likelihood function – Equation S29) show a relatively minor impact of the linear assumption.

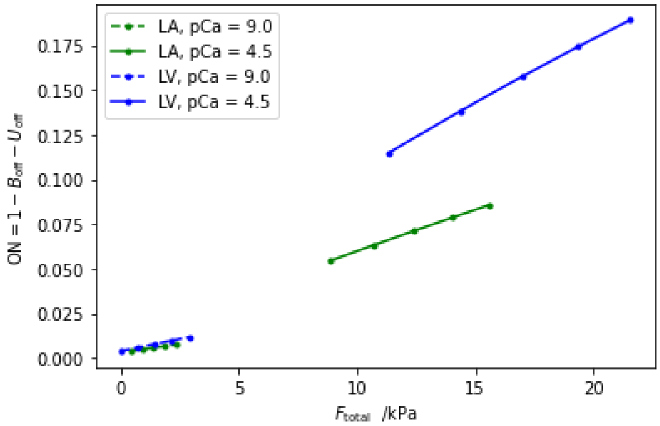

Figure S4. Contributions of active and passive force to cross-bridge recruitment. The proportion of cross bridges in the ON state is plotted as a function of total force under conditions of low (pCa $=$ 9.0) and high calcium (pCa $=$ 4.5). Gradients are $d\text{ON}/dF = 1.83 \times 10^{-3}$ kPa^{-1} for LA and 2.74×10^{-3} kPa^{-1} for LV at pCa 9.0; 4.65×10^{-3} kPa^{-1} for LA and 7.25×10^{-3} kPa^{-1} for LV at pCa 4.5.

therefore non-trivial and remained to be established. We sought to address this question by considering jointly the model parameters associated with the passive and active forces.

To demonstrate the respective contributions to OFF/ON dynamics of the passive and active forces, we computed the proportion of ON states in the isometric steady state, quantified as $\mathrm{ON} = 1 - B_{\mathrm{off}} - U_{\mathrm{off}}$. We compared the length dependence of ON under low and high [Ca^{2+}] conditions. SL was varied from 1.6 to 2.3 μm to cover a range of passive and active force. The results, plotted in Figure S4, show a steeper gradient $d\mathrm{ON}/d\mathrm{SL}$, greater by a factor of $\sim$ 2.6, for pCa = 4.5 compared to pCa = 9.0, for both LA and LV. This result indicates that, while passive force is a necessary ingredient for initiating LDA, active force plays an important role in controlling the effect quantitatively. (See Discussion in the main manuscript.)

S7.3 Identifying the main contribution to the energy cost of isometric force generation

The value of the energy cost thus hinges primarily on two quantities: $S_{\mathrm{ss}}$and k_{SU}. To identify which of these most significantly accounts for the LA/LV difference, we generated hybrid models where, starting from each calibrated model, we sequentially substituted the S_{ss} and k_{SU} values from the other model, while monitoring the corresponding impact on C. We proceed as follows.

Given the identities

$$
\begin{aligned}
C &= S_{\mathrm{ss}} k_{\mathrm{SU}} / F^{\mathrm{ss}}_{\mathrm{a,max}} && \text{(S33)} \\
S_{\mathrm{ss}} &= U_{\mathrm{ss}} \times r_{\mathrm{S}} / (1 - r_{\mathrm{S}})(1 - r_{\mathrm{W}}) && \text{(S34)} \\
k_{\mathrm{SU}} &= k_{\mathrm{WS}} \times r_{\mathrm{W}} (1/r_{\mathrm{S}} - 1) && \text{(S35)}
\end{aligned}
$$

we swapped the S_{ss} values (to a sufficient approximation) between LA and LV by swapping r_{W} and r_{S} while also adjusting k_{WS} to keep k_{SU} fixed and keeping all other model parameters unchanged. Next, we swapped k_{SU} by scaling k_{WS} while keeping r_{W} and r_{S} constant, which keeps S_{ss} fixed. The changes in force cost resulting from each swap were computed after each of these substitutions, as shown in Figure S5(c).

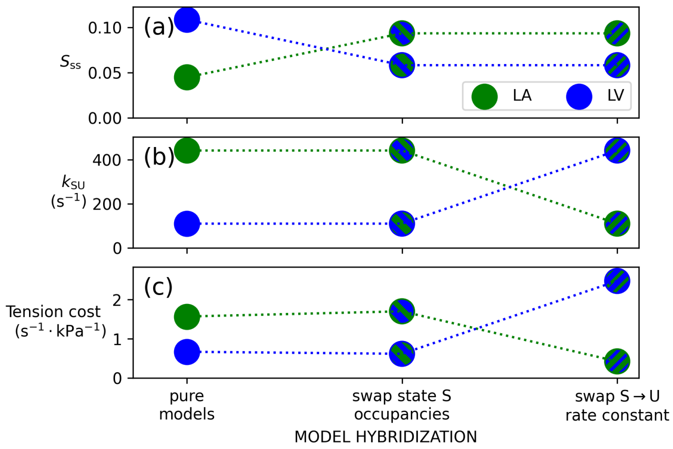

Figure S5. Comparing the contributions to the energetic cost of isometric force. We investigated which contribution more significantly to the force cost (Equation S33): the occupancy S_{ss} of the strongly bound crossbridge state, or the rate constant k_{SU} of crossbridge detachment. Starting from the calibrated models, hybrid models were generated, sequentially substituting (a) S_{ss}, (b) k_{SU} while monitoring (c) the resulting force cost $C = S_{\mathrm{ss}} k_{\mathrm{SU}} / F^{\mathrm{ss}}_{\mathrm{a,max}}$.

References

[1] Chris W Patton, Stuart Thompson, and David Epel. Some precautions in using chelators to buffer metals in biological solutions. *Cell Calcium*, 35(5):427–431, 2004.

[2] Sander Land, So-Jin Park-Holohan, Nicolas P Smith, Cristobal G. dos Remedios, Jonathan C Kentish, and Steven A Niederer. A model of cardiac contraction based on novel measurements of tension development in human cardiomyocytes. *Journal of Molecular and Cellular Cardiology*, 106:68–83, 2017.

[3] Alexandre Lewalle, Gregory N Milburn, Kenneth S Campbell, and Steven A Niederer. Cardiac length-dependent activation driven by force-dependent thick-filament dynamics. *Biophysical Journal*, 123:1–14, 2024.

[4] David J C MacKay. *Information Theory, Inference, and Learning Algorithms.* Cambridge University Press, 7.2 edition, 2005.

[5] J Goodman and J Weare. Ensemble Samplers With Affine Invariance. *Communications in Applied Mathematics and Computational Science*, 5(1):65–80, 2010.

[6] Daniel Foreman-Mackey, David W. Hogg, Dustin Lang, and Jonathan Goodman. emcee : The MCMC Hammer . *Publications of the Astronomical Society of the Pacific*, 125(925):306–312, 2013.

[7] Emi Tanaka and Francis K C Hui. Symbolic Formulae for Linear Mixed Models. In Hien Nguyen, editor, *Statistics and Data Science (Research School on Statistics and DataScience, Melbourne, Australia, July 24-26, 2019)*. Springer Singapore, 2019.

[8] Sam Coveney, Cesare Corrado, Jeremy E. Oakley, Richard D. Wilkinson, Steven A Niederer, and Richard H. Clayton. Bayesian Calibration of Electrophysiology Models Using Restitution Curve Emulators. *Frontiers in Physiology*, 12(July):1–16, 2021.

[9] Andrea Saltelli, Paola Annoni, Ivano Azzini, Francesca Campolongo, Marco Ratto, and Stefano Tarantola. Variance based sensitivity analysis of model output. Design and estimator for the total sensitivity index. *Computer Physics Communications*, 181(2):259–270, 2010.

[10] Stefano Longobardi, Anna A. Sher, and Steven A Niederer. *In Silico Mapping of the Omecamtiv Mecarbil Effects from the Sarcomere to the Whole-Heart and Back Again*, volume 12738. Springer International Publishing, 2021.

[11] Allan J Brady. Length dependence of passive stiffness in single cardiac myocytes. *American Journal of Physiology - Heart and Circulation Physiology*, 260(4):H1062–1071, 1991.

[12] Daniel Fish, Jan Orenstein, and Sherman Bloom. Passive Stiffness of Isolated Cardiac and Skeletal Myocytes in the Hamster. *Circulation Research*, page 267, mar 1984.
